# Erythrocyte Aging, Protection via Vesiculation: An Analysis Methodology via Oscillatory Flow

**DOI:** 10.3389/fphys.2018.01607

**Published:** 2018-11-16

**Authors:** Robert J. Asaro, Qiang Zhu, Pedro Cabrales

**Affiliations:** ^1^Department of Structural Engineering, University of California, San Diego, San Diego, CA, United States; ^2^Biological Engineering, University of California, San Diego, La Jolla, CA, United States

**Keywords:** vesiculation, oscillatory flow, oxidative damage, erythrocyte vesicles, self protection

## Abstract

We demonstrate that erythrocyte deformations, specifically of a type as occur in splenic flow (Zhu et al., 2017), and of the type that promote vesiculation can be caused by simple, yet tailored, oscillatory shear flow. We show that such oscillatory shear flow provides an ideal environment to explore a wide variety of metabolic and biochemical effects that promote erythrocyte vesiculation. Deformation details, typical of splenic flow, such as *in-folding* and implications for membrane/skeleton interaction are demonstrated and quantitatively analyzed. We introduce a theoretical, essentially analytical, vesiculation model that directly couples to our more complex numerical, multilevel, model that clearly delineates various fundamental elements, i.e., sub-processes, that are involved and mediate the vesiculation process. This analytical model highlights particulary important vesiculation precursors such as areas of membrane/skeleton disruptions that trigger the vesiculation process. We demonstrate, using flow cytometry, that the deformations we experimentally induce on cells, and numerically simulate, do not induce lethal forms of cell damage but do induce vesiculation as theoretically forecasted. This, we demonstrate, provides a direct link to cell membrane/skeletal damage such as is associated with metabolic and aging damage. An additional noteworthy feature of this approach is the avoidance of artificial devices, e.g., micro-fluidic chambers, in which deformations and their time scales are often unrepresentative of physiological processes such as splenic flow.

## 1. Introduction and background

Without a nucleus, a mature erythrocyte (or RBC) contains a cytosol enclosed within a highly flexible cell membrane. This composite membrane, consisting of a lipid bilayer supported by a membrane skeleton, is essential to its structural integrity and stability. The basic picture is that of a skeleton created from junctional complexes (JCs) bound to each other via head-to-head associations of spectrin (Sp), which is anchored to the fluidic lipid bilayer at linkage sites (Mohandas and Evans, 1994; Mohandas and Gallager, 2008; Lux, 2015). RBCs possess one of the best characterized molecular architectures among all cell types and are thus often chosen as a model system to study various functions of cells, especially those that involve mechanical behavior and response. A particularly intriguing process that erythrocytes exhibit is vesiculation, during which part of the phospholipid bilayer separates from the skeleton and the rest of the membrane to create a vesicle (Peng et al., 2010; Zhu et al., 2017). The occurrence of vesiculation depends on the magnitude of the skeleton-bilayer dissociation stress—that develops during deformation - as well as the strength of the skeleton-bilayer connectivity—which may be compromised during the aging process as discussed below. To date there is incomplete knowledge about the detailed process or physiological significance of vesiculation although evidence has mounted as to its role in erythrocyte aging (Fox et al., 1991; Schwarz-Ben Meir et al., 1991; Glaser et al., 1994; Willekens et al., 2003b; Hattangadi and Lodish, 2007; Bosman et al., 2012; Zhu et al., 2017). Herein we present a novel methodology for a coupled experimental-theoretical study of human erythrocyte vesiculation.

**Figure 1:**
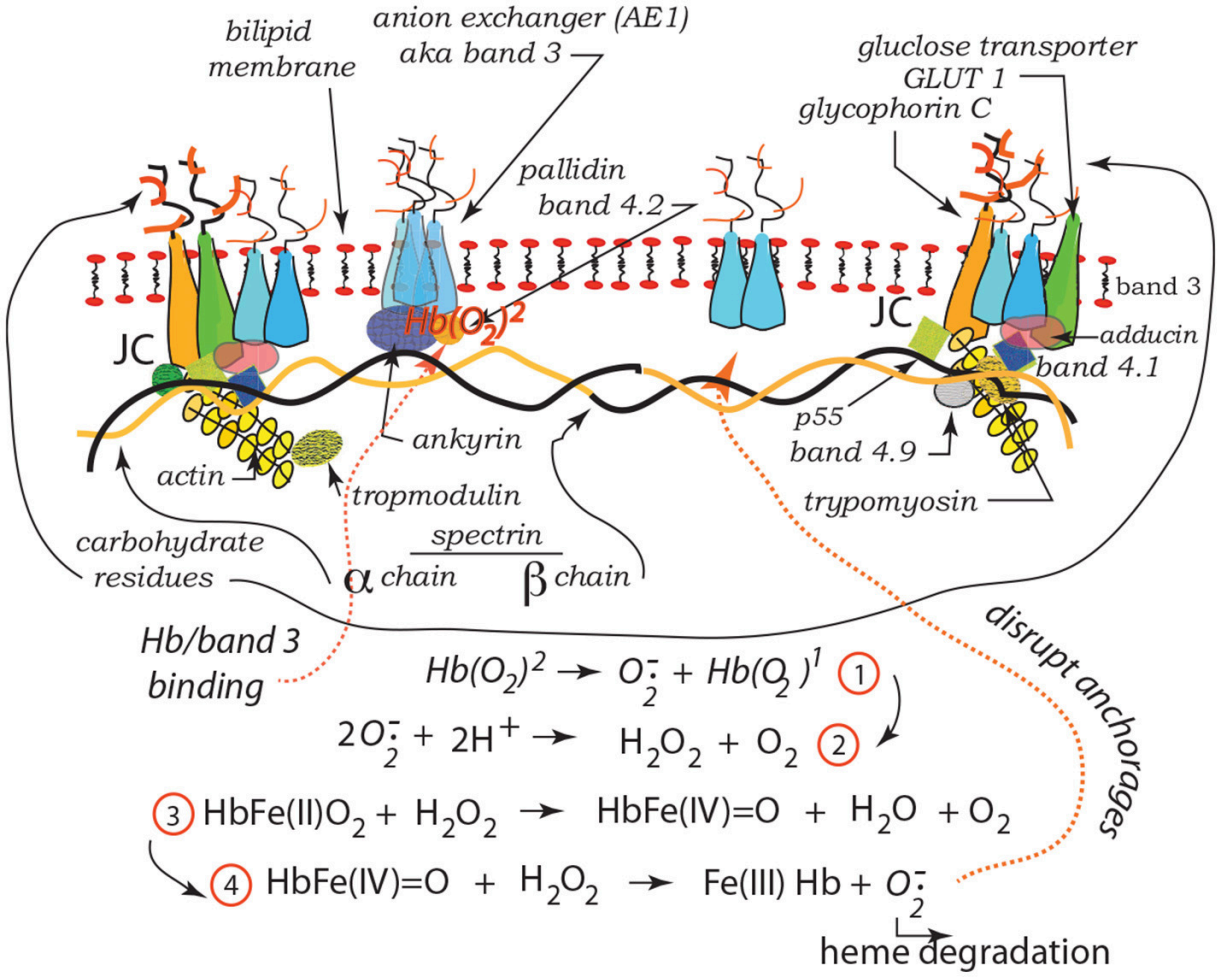
Hb binding to band 3. ROS reactions leading with Hb to degraded heme and binding and disruption of band 3 skeleton anchorage (Low et al., 1985; Nagababu and Rifkind, 2000; Cao et al., 2009; Rifkind and Nagababu, 2013; Mohanty et al., 2014). The JC complex is described in section 2 in detail.

Our recent studies have demonstrated the strong prospects for human erythrocyte vesiculation during flow through the spleen (Zhu et al., 2017). Our numerical simulations, along with our underlying analysis of skeleton-membrane interactions provided vital insight into the coupling of the RBC aging process with metabolic processes that occur during the aging process. These aging processes include, among others, denaturing of hemoglobin, including such occurring via oxidative damage, and binding of denatured Hb to the skeleton-membrane attachment sites causing disruption of the skeleton-membrane attachment (Willekens et al., 2003a,b; Bosman et al., 2012). Erythrocyte aging, involving vesiculation, in turn involves processes such as oxidative stress (ROS) and other reactive stresses such as involves, *inter alia*, nitrates (NOS), phenyldrazine, *Ca*^2+^ uptake, or Cd. Figure 1 illustrates examples of various key steps of oxidative stress and damage via ROS (Low et al., 1985; Fox et al., 1991; Nagababu and Rifkind, 2000; Cao et al., 2009; Rifkind and Nagababu, 2013; Mohanty et al., 2014). Such effects can be readily explored via our developed methodology as they contribute to vesiculation (Willekens et al., 2003a; Cao et al., 2009; Rifkind and Nagababu, 2013; Mohanty et al., 2014; Zhu et al., 2017).

In the context of splenic flow, our simulations led to a description of what is most likely required to induce vesiculation during splenic flow and provided a paradigm that, in fact, supported the view that erythrocyte vesiculation, including vesiculation during splenic flow, may be a *self-protective* mechanism (Willekens et al., 2003b; Bosman et al., 2012; Zhu et al., 2017). In this context, self-protection involves the elimination of *removal molecules* such as denatured Hb as well as phosphatidylserine (PS) and IgG that are known to be associated with cell removal (Willekens et al., 2003b; Bevers and Williamsonl, 2010; Wieschhaus et al., 2012; Kostova et al., 2015; Bevers and Williamson, 2016; Bevers et al., 2017). Our results, in addition, revealed that as vesiculation occurs, presumably in younger deformable cells, and hemoglobin concentration increases and membrane area decreases, the prospects for vesiculation decreases; hence the self-protective mechanism may be effectively shut off with aging. This is closely linked with a loss in cell deformability that is often related to a loss in cell viability. In addition, our methods should be expected to shed new light on the effects of oxidative damage, caused by reactive oxidative species (ROS), on the vesiculation process (Hattangadi and Lodish, 2007; Marinkovic et al., 2007). Thus, the continued study of the vesiculation process is warranted as it appears so closely tied to cell aging, to cell viability, and cell death. Particularly important is to directly link vesiculation to the vital factors of aging, such as those associated with oxidative damage and a methodology to confirm the various hypotheses of the mechanisms involved.

### 1.1. Background on extracellular vesicles: viz. microvesicles (MV's)

The extracellular space of multicellular organisms contains a variety of species including, *inter alia*, metabolites, ions, proteins, polysaccharides, *etc*. and also a large number of mobile membrane bound vesicles that have been collectively referred to as *extracellular vesicles* (EV's) (Morel et al., 2010; György et al., 2011; Raposo and Stoorvogel, 2012). There are ongoing attempts at classification of EV's where distinctions are based on, for example, size, constituency, and mechanisms of formation (Morel et al., 2010; György et al., 2011; Raposo and Stoorvogel, 2012; Alaarg et al., 2013). For example, *exosomes* are generally placed in a size range with diameters <100 nm whereas *microvesicles* (MV's) are generally placed in the diameter range of 100-1,000 nm. Moreover, exosomes are generally created intracellularly and excreted, whereas MV's are formed through budding from the bilipid membrane. Exceptions, however, may exist as we note, for example, the report by Booth et al. (2006) of exosomes being in the size range 50-100 nm budding from T cells. Herein we focus on what we call (following e.g., Morel et al., 2010; György et al., 2011; Raposo and Stoorvogel, 2012; Alaarg et al., 2013), MV's budded from erythrocyte membranes, and generally expected to be in the size range 100–250 nm. Our analysis, however, does not preclude budded vesicles in a size range <100 nm, yet probably not smaller than 40-50nm as discussed below.

MV formation is associated with structural alterations of the bilipid membrane and a host of factors that disrupt erythrocyte skeleton-membrane attachment (Lutz et al., 1977; Willekens et al., 2003a,b; Morel et al., 2010; György et al., 2011; Bosman et al., 2012; Raposo and Stoorvogel, 2012; Alaarg et al., 2013). This we specifically address herein. Causes of disruption include, *inter alia*, various forms of oxidative damage associated with aging (Willekens et al., 2003a,b; Hattangadi and Lodish, 2007; Bosman et al., 2012), nitrite induced stress, i.e., NOS (Nagababu and Rifkind, 2000; Cao et al., 2009; Rifkind and Nagababu, 2013), excess Ca^2+^ uptake (Allan and Mitchell, 1977; Dodson et al., 1987; Pasquet et al., 1996; Bratosin et al., 2001; Chunyi et al., 2001; Klarl et al., 2006), and ATP depletion (Lutz et al., 1977). Association of increased MV production with diseases such as hemolytic anemias has been reported and discussed (Alaarg et al., 2013). Moreover, although we demonstrate that imposed deformation on cells, such as occurs e.g., in splenic flow, can indeed promote vesiculation the known phenomenology shows that intense imposed deformations are not required for budded vesicles to form. Indeed, Fox et al. (1990, 1991) have shown that disruption of the actin based JC of the skeleton promotes microvesiculation without imposed shear deformation. In addition, Schwarz-Ben Meir et al. (1991) and Glaser et al. (1994) have shown that calpain (Ca^2+^-dependent thiol protease) binds to, and degrades, band 3 that is a major erythrocyte membrane attachment protein for the skeleton; this leads to membrane loss via vesiculation. These two studies were particularly concerned with aging, not just of the red blood cell, but of people over 70 years of age in whom the effects were accelerated. Still again, Kostova et al. (2015) have recently discussed how the Ca^2+^ ionophore ionomycin provides an effective pathway, due to membrane disruption, to vesiculation. Wieschhaus et al. (2012) report of the effects of increased Ca^2+^ uptake in degrading attachment proteins such as band 3, 4.1, and ankyrin in the presence of calpain-1 and consequently reducing cell deformability. These, among other observations, provide a basis for our model of budding that begins with localized, i.e., small 20–40 nm, sections of the membrane whose attachments to the skeleton have been disrupted. *Specifically, we study herein how vesiculation can be explored using a remarkably simple imposed oscillatory shear flow that allows for readily tailored modes and intensities of deformation*.

Finally, we note that microvesicles budded from erythrocytes are typically characterized by exposure of PS on their outer bilayer leaflet (Dasgupta et al., 2009; Bevers and Williamsonl, 2010; Bevers and Williamson, 2016; Bevers et al., 2017). Hence translocation of PS from the inner-to-outer leaflets may be viewed as a part of the vesiculation process or, in fact, a contributor to vesiculation. Two possible pathways for a PS translocation role are: (i) it induces a shape change involving initial curvature of the membrane out from the skeleton (Sheetz and Singer, 1974), and/or (ii) that it acts as an energy source as does skeletal deformation (Sheetz and Singer, 1974; Bevers and Williamson, 2016). Lipid asymmetry represents stored free energy, given the work required to create it against a steep concentration gradient, that can be made available to induce deformation and aid the vesiculation process as described below in section 4.2. Also as we show in section 4.3, pre-curvature provides a “boost” in the membranes bulging process by which a vesicle forms. This view is supported by Bevers et al. (2017), who demonstrated that in RBCs affected by Scott's syndrome microvesiculation was diminished in frequency (see also Lhermusier et al., 2011). Dasgupta et al. (2009) studied microvesiculation in mice treated with lactadherin and showed a lactadherin deficiency led to increased numbers of microvesicles at steady state.

### 1.2. Background on recent theoretical innovations

Our simulations (Zhu et al., 2017) of erythrocyte flow through simulated venous-like slits of the spleen were performed with a hierarchical model that followed the cell's deformation process in detail. Noteworthy is that this model considers the cell membrane and skeleton separately and explicitly accounts for the lateral mobility of the skeleton's attachment trans-membrane proteins through the membrane. Hence during deformation, the areal density of skeletal attachments may change, viz. decrease due to large areal deformation of the skeleton, and thereby weaken the overall skeleton-membrane attachment as described in detail elsewhere (Peng et al., 2010; Zhu et al., 2017). However, as the trans-membrane attachment proteins have finite (but documented) mobility, the process of changing skeletal density has its own time scale that operates within the deformation's time scale; this too was predicted (see Zhu et al., 2017 and its discussion of the results of Peng et al., 2010, 2011; Peng and Zhu, 2013). Now, in this we find that deformation processes in splenic flow, such as what we have dubbed *in-folding*, produce a “tension” (dubbed *negative pressure* in Zhu et al., 2017) between the skeleton and membrane that can promote separation that leads to vesiculation (Zhu et al., 2017). However, a key feature of this is that the time scales of splenic flow are such that large changes (decreases) in the areal density of attachment points are unlikely (Zhu et al., 2017). Hence the vital role of attachment disruption via, e.g., binding of denatured Hb, is required. *That is, since areal density reduction of attachments does not occur in time during splenic flow deformation restructuring, it must occur by metabolically induced disruption of anchorage points*.

Our simulations were quite detailed as to what was required with respect to such disruptions to cause separation. But to study vesiculation by inducing erythrocyte flow through artificial slits (as can be made by pores or slits in commercial filters can be) however, problematic. For example, flow through artificial slits in the splenic range of 1 μm generally leads to cell damage and fragmentation under very different time scales. This brings us to the following novel discovery, prospect and proposal.

*We have found, that under certain ranges of frequency and shear amplitudes, simple oscillatory shear flow causes cell deformations whose characteristics (e.g., magnitude of shear deformation and even in-folding) and time scales are comparable to those in splenic flow, and thereby may induce vesiculation*. This provides the prospect of following vesiculation without the use of artificial slits or resorting to deformation methods such as micro-pipette aspiration — that, as it happens, we have already shown can cause vesiculation but whose time scales are quite unlike those of splenic flow. Hence we have discovered the prospect of studying vesiculation in an open environment, devoid of artificial structure, e.g., the slits or pores of man-made filters, in which control of chemistry is readily achievable. This is now illustrated by way of past and new results specific to our modeling in sections 3 and 4.

## 2. Overview of the simulation model and results

One of the most severe physiological deformations a RBC sustains occurs inside the spleen, where it “squeezes” through slits as narrow as 0.6 micron (Chen and Weiss, 1973; Mebius and Kraal, 2005; Lux, 2015; Zhu et al., 2017). Under this extreme condition the cell undergoes dramatic shear variations, including a novel deformation mode called *infolding* discovered in recent computational studies (Freund, 2013; Salehyar and Zhu, 2016; Zhu et al., 2017). The mechanical loads on the cell membrane include the external loads (traction and normal stress) from the external/internal fluids, and the internal stresses within the membrane itself. Hereby the internal stresses are categorized into two parts, *viz*. in-plane and out-of-plane. The in-plane stresses contain an isotropic component, a shear component, and friction between the skeleton and the bilayer. The out-of-plane stress is the normal interaction stress between the skeleton and the bilayer that either pulls them together (association) or pushes them apart (dissociation). No prestress in the skeleton is considered so that the stress-free state of the cell coincides with its natural biconcave state. The membrane thus possesses shape memory.

**Figure 2:**
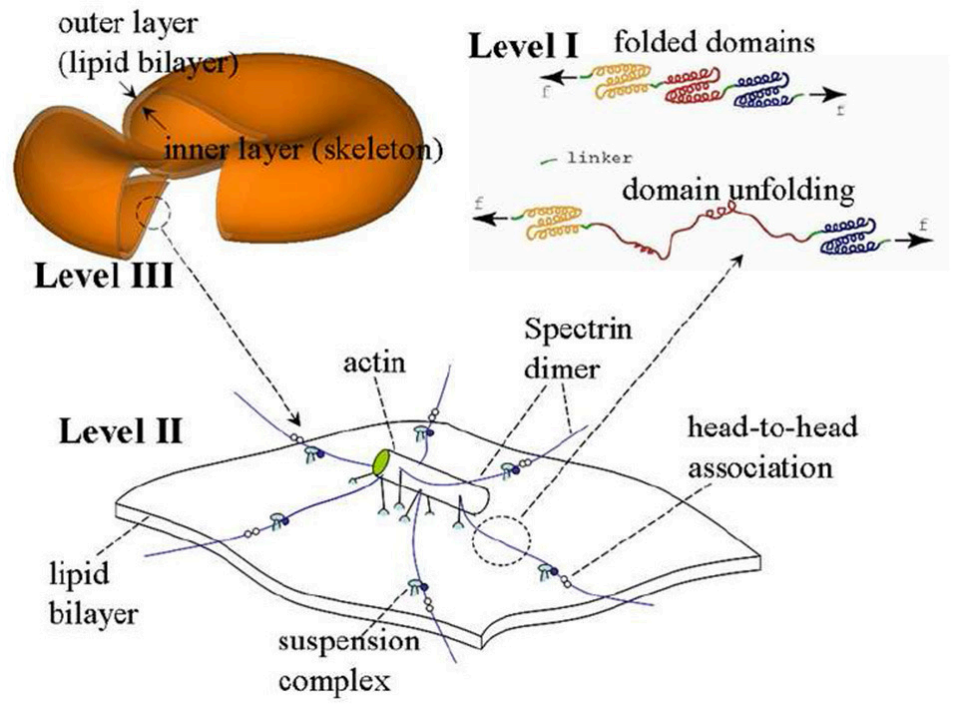
A sketch of the multiscale RBC model.

The model we use is based on a multiscale multi-physics framework as described in detail elsewhere (Zhu et al., 2007, 2017; Zhu and Asaro, 2008; Peng et al., 2010, 2011; Peng and Zhu, 2013). As illustrated in Figure 2, this approach includes three models at different scales: in the complete cell level (Level III) the membrane is modeled as two layers of continuum shells using the finite element method; the constitutive properties of the inner layer (the membrane skeleton) are obtained from a 3D molecular-detailed JC model (Level II); the mechanical properties of Sp, including its folding/unfolding reactions (Bustamante et al., 1994; Lee and Discher, 2001; Zhu and Asaro, 2008), are obtained with a stress-strain model based on Arrhenius equation (Level I). The fluid-cell interaction is mathematically formulated within a low-Reynolds number Stokes/Oseen flow framework and solved using a boundary-element method (for details see Appendix).

**Figure 3:**
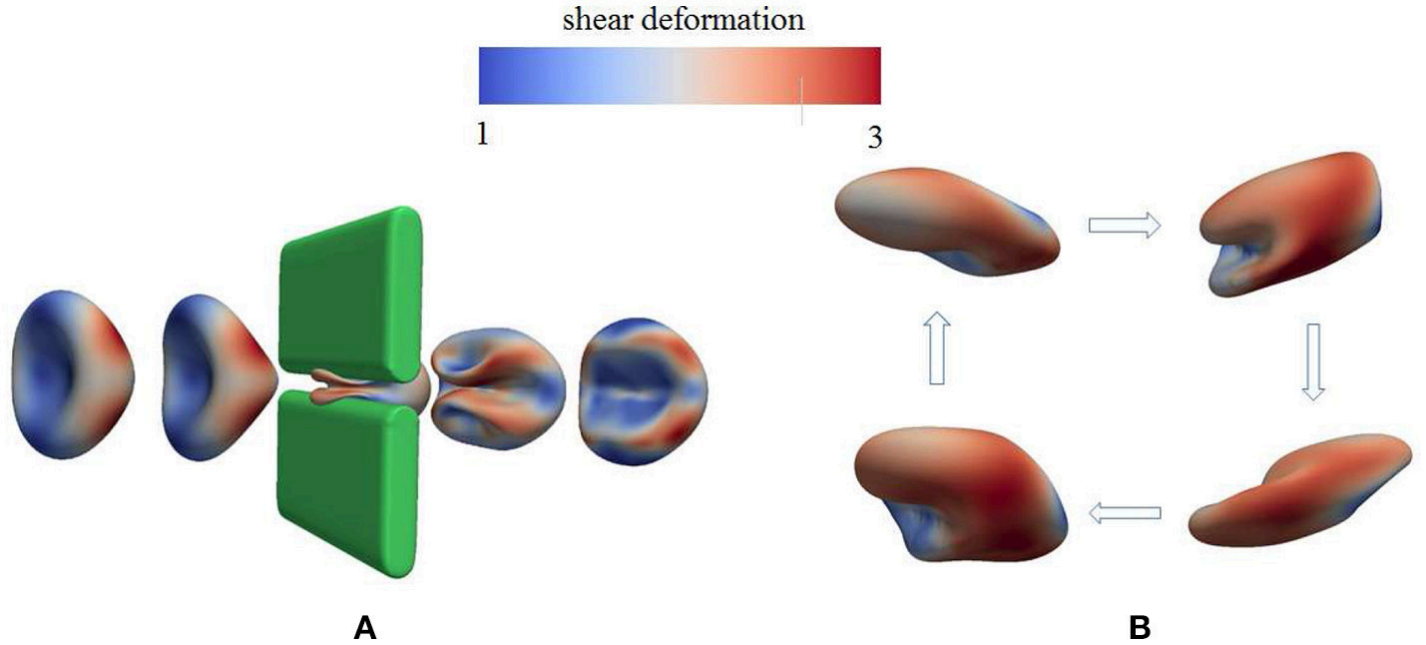
Occurrence of membrane infolding in **(A)** splenic flow (Salehyar and Zhu, 2016) and **(B)** oscillatory shear flow. In **(B)** the cytosolic/medium viscosity ratio was 1; the average shear stress applied to the cell was in the range $\bar{\tau }\sim 6Pa$. The peak shear rate is 1,000 s^−1^, and the frequency is 67 Hz. In both figures the color contour corresponds to shear deformation χ (see section 3.2 for the definition of χ) of the skeleton with the value ranging from 1 (in blue) to 3 (in red). χ = λ_1_/λ_2_ with λ_*i*_, *i* = 1, 2 being the principal skeletal stretches (see section 3.2).

As demonstrated in Figure 3A (Salehyar and Zhu, 2016; Zhu et al., 2017), under certain conditions right after the cell passes through the slit a part of its membrane bends inward to form a concave region on its rear surface. Our model also illustrates that due to the increased surface curvature and skeletal energy density, infolding usually coincides with significantly increased dissociation stress between the skeleton and the lipid bilayer. The values of stress we found are orders of magnitude higher than those in normal conditions such as in a steady shear flow field (Zhu et al., 2017).

Further examination shows that in addition to splenic flow, infolding may happen in other scenarios, e.g., in an oscillatory shear flow field. As shown in Figure 3B, when a RBC is put in a shear flow field with sinusoidally varying shear strength and direction, it undergoes similar infolding behavior, resulting in increased skeleton-bilayer dissociation stress inside the membrane. Indeed, our simulations suggest that within the range of parameters achievable in laboratory conditions, this dissociation stress in oscillatory shear flows attains levels comparable to splenic flow and may thus contribute to vesiculation (see also **Figure 7**). Meanwhile, the deformations of the cell remain moderate so that the risk of cell bursting is minimal.

### 2.1. Examples of oscillatory flow

**Figure 4:**
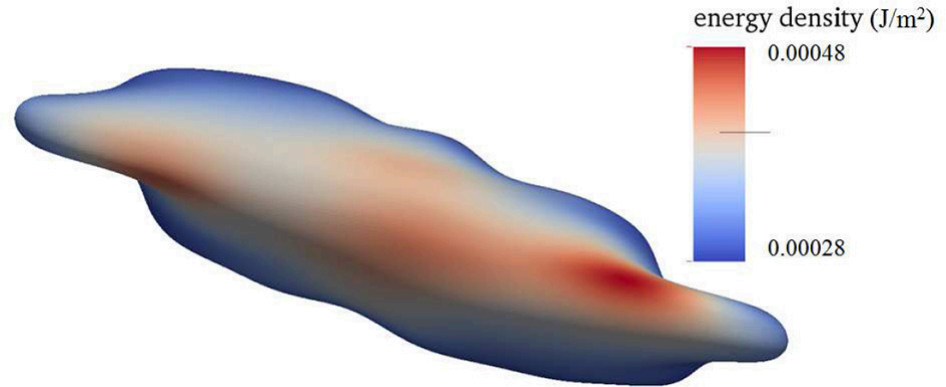
Contours of skeleton energy density, χ, in an oscillatory shear flow field. The shear rate was σ = 2,000s^−1^ at a frequency of 100 Hz. The cytosolic/medium viscosity ratio was equal to 1; the average shear stress applied to the cell was in the range $\bar{\tau }\sim 12Pa$. Areas in red color are judged to be those with a high probability for vesiculation (see section 4.4).

Figure 4 shows a snapshot of the deformed cell subject to a shear flow field characterized by frequency 100 Hz with a shear rate σ = 2,000*s*^−1^. The contours shown are for skeleton deformation energy density as so labeled with the insert. We note that in this case the maximum skeleton energy density is nearly 5 × 10^−4^Jm^−2^ and is located on **Figure 10** as point “a”; this is used in later discussion of vesiculation in section 4.4.

**Figure 5:**
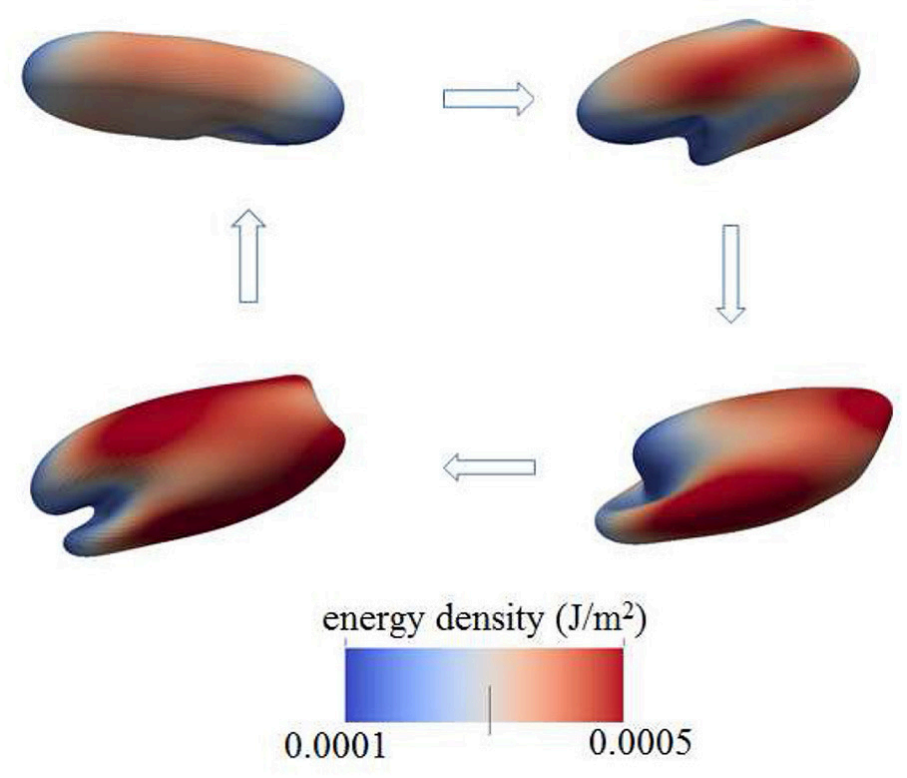
Contours of skeleton energy density, χ, in an oscillatory shear flow field. The shear rate was σ = 5,000*s*^−1^ at a frequency of 67 Hz. The cytosolic/medium viscosity ratio is 5; the average shear stress applied to the cell was in the range $\bar{\tau }\sim 6Pa$. The energy densities range from about 1 × 10^−4^Jm^−2^ (blue)−5 × 10^−4^Jm^−2^ (red).

**Figure 6:**
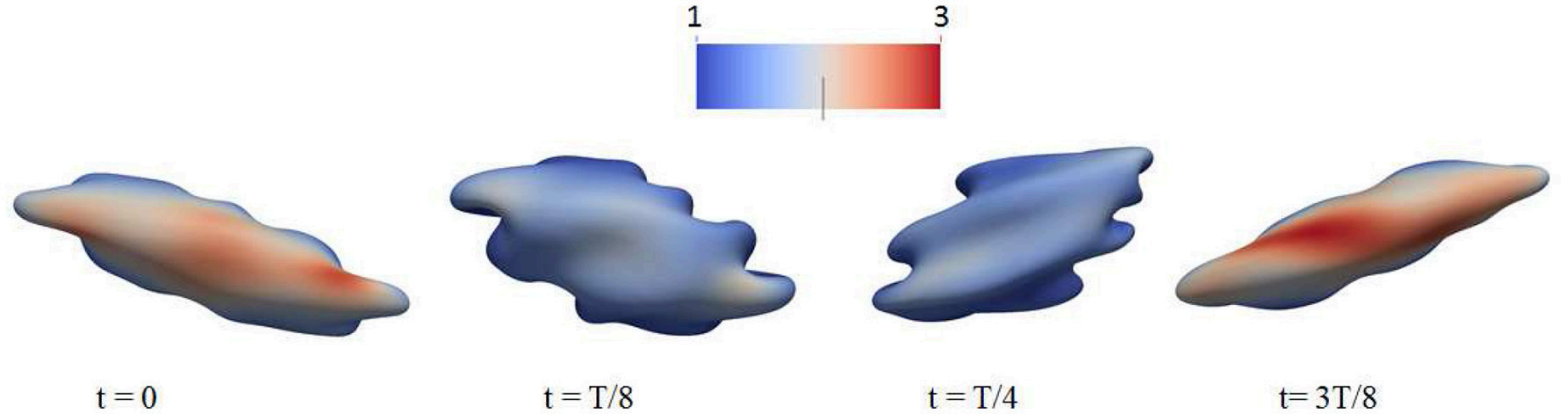
Deformation of a red blood cell in a strong high-frequency oscillatory shear flow field. The amplitude of the shear flow is 2,000 s^−1^ and the frequency is 100 Hz. The cytosolic/medium viscosity ratio was equal to 1; the average shear stress applied to the cell was in the range $\bar{\tau }\sim 12Pa$. The color contour shows the shear deformation χ of the skeleton. Areas in red color are judged to be those with a high probability for vesiculation (see section 4.4). χ = 1 (blue) to 3 (red).

Still another case is shown is shown in Figure 5 where in this case the shearing rate is well above physiological rates and is set at σ = 5,000*s*^−1^; the frequency of flow was 67 Hz. This case differs from those also shown, i.e., in Figures 3, 4, 6 in that a higher viscosity is prescribed for the cell interior. In this case, the contours are of skeleton shear deformation and like those for Figures 3, 6 the area deformations are just above unity. That means that the maximum skeletal energy density is near to point “b,” close to point “a,” of Figure 10.

### 2.2. Additional perspective on oscillatory shear flow

We believe this ability to tailor deformation via oscillatory flow presents a novel methodology for probing the metabolic effects of membrane/skeleton degradation that defines aging. For this reason we have run additional simulations to investigate the response of red blood cells to strong oscillatory shear flows. This has uncovered additional interesting phenomena. For example, in Figure 6 we plot the deformation of a cell to a sinusoidally varying shear flow of 100 Hz and peak shear rate of 2,000 s^−1^. It is seen that the cell undergoes complicated deformations with multiple infolded regions on its membrane. These regions are characterized by large curvature as well as significant dissociation stress between the cytoskeleton and the lipid bilayer. Indeed, our simulations indicate that in cases like these the dissociation stress reaches levels of O(100)Pa, comparable to the level that can be reached as the cell passes through inter-endothelial slits in a spleen (Zhu et al., 2017). This provides evidence that, in terms of skeleton-bilayer dissociation, an oscillatory flow field is able to create conditions similar to the physiological conditions of splenic flow.

Furthermore, we carefully monitored maximal areal deformations of the lipid bilayer during these processes and note that even under extreme conditions, such as the case of Figure 6, they were only ~1−2%; this is significantly smaller than the approximate areal strain of 10% a cell can sustain under dynamic (i.e., impact) conditions (Li et al., 2013). It is also at, or below, the somewhat lower values in the range 2–3% found under different deformation conditions (Leverett et al., 1972; Sandza et al., 1974; Sutera and Mehrjardi, 1975; Evans et al., 1976; Sutera et al., 1977; Daily et al., 1984; Watanabe et al., 2006; Wantanbe et al., 2007; Meram et al., 2013; Hashimoto, 2014; McNamee et al., 2016; Horobib et al., 2017). We thus conclude that pressure-induced cell bursting due to large area deformation of the bilayer is not likely to occur in these conditions as discussed further in section 4.5 along with additional detail and perspective.

### 2.3. Vesiculation forecasts with oscillatory flow

**Figure 7:**
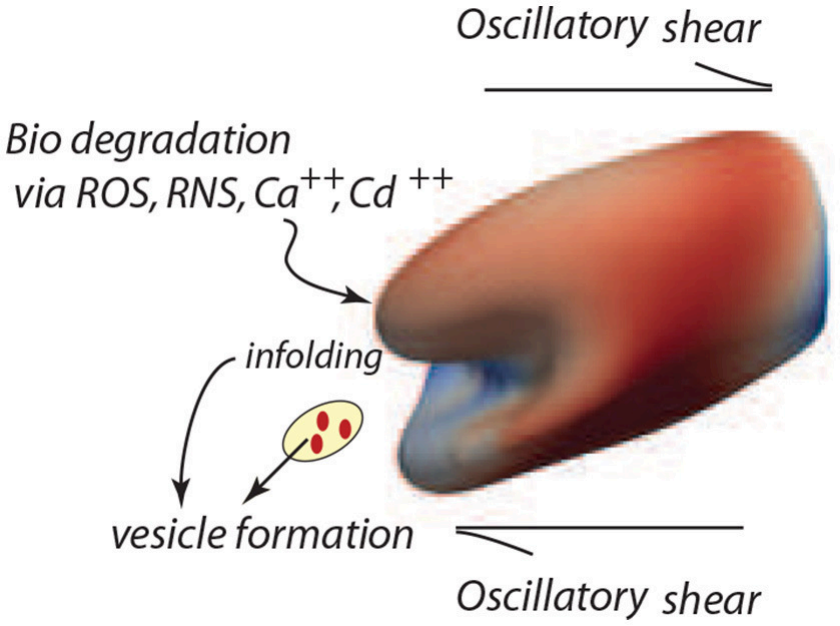
Degradation of the cell's membrane/skeleton attachment structure induces vesiculation under the deformations of oscillatory shear flow as in the case of Figure 3B (upper right frame).

Based on the analysis of our next section, based in turn on our simulation results of cell deformations presented in this section, we suggest that areas shaded in red and zones of in-folding as shown, for example, in Figure 7 are most probable vesiculation sites. The areas in red generally experience skeletal energy densities in the range $\epsilon_{0}\sim 3-5\times 10^{-4}{\text{Jm}}^{-2}$ and shear stresses in the range $\bar{\tau }\sim 5-10$Pa that as discussed in sections 3, 4.4, and 4.5 promote vesiculation.

## 3. Prospects for vesiculation

The model developed herein focuses on the mechanisms of vesiculation as a natural, essential, mechanistic component of the erythrocyte aging process. However, in a more general context the focus is on cellular aging *per se*, and on the role of oxidative stress and other biochemical stress on aging (Iuchi et al., 2007; Pandey and Rizvi, 2010, 2011; Mohanty et al., 2014). Such aging effects are manifest in at least two ways, viz. a weakening of the skeletal/bilayer connection as measured by γ and in the size of the initial skeletal/bilayer separation. The particular contribution here is that we provide a detailed, quite specific, yet quite simple, mechanistic pathway by which erythrocyte aging occurs and by which erythrocytes are removed. This process involves a general metabolic aging via ROS and other biochemical activity and in this way its analysis provides still another clue as to the effects of biochemical activity on the aging process (Dröge, 2002; Rattan, 2006; Harman, 2009; Tsuda, 2010). Our unique approach follows vesiculation as promoted by biochemical aging and the imposition of splenic-like deformations using our novel tool of oscillatory shear flow as illustrated in Figure 7.

### 3.1. Vesiculation model

**Figure 8:**
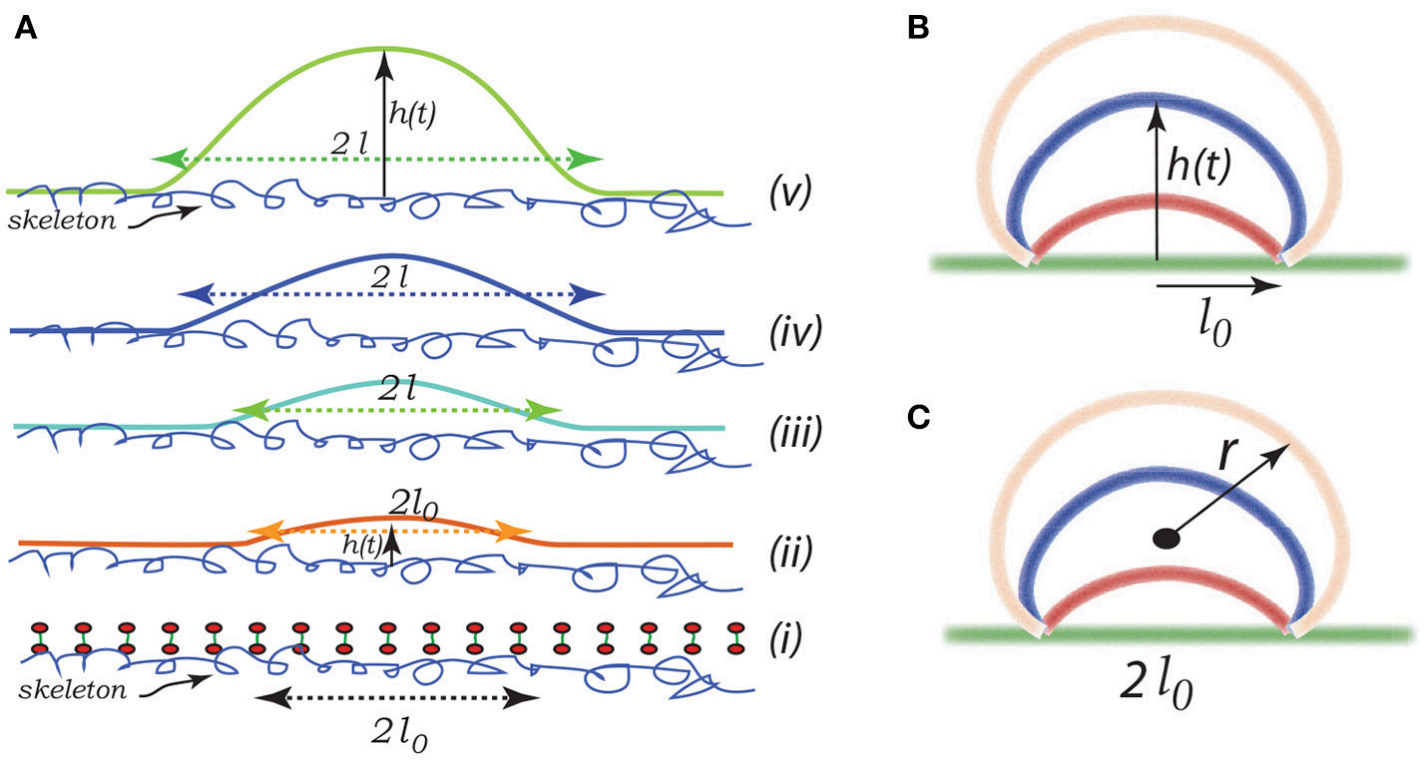
Schematic of a budding vesicle. In **(Aii)** we illustrate a pre-curvature as discussed in the text. From **(Aiii** to **Av)** we have progressing membrane bulging leading to vesiculation. **(B,C)** depict a model expansion analyzed below. Vesiculation begins within a damaged membrane/skeleton area of diameter 2ℓ_0_. Driven by release of stored skeletal energy a bulge forms and possibly grows as in **(B,C)** where the energy pathway is given in Figure 11. The final stage of pinching-off and vesicle release in illustrated in Figure 12.

Imagine that initially a quite localized circular patch of membrane, of radius ℓ_0_, separates from the skeleton, as in the bottom most figure, Figure 8Ai. We can imagine that this occurs due to the binding of Hb (Willekens et al., 2003a,b; Bosman et al., 2012), or by oxidative damage (Rifkind and Nagababu, 2013; Mohanty et al., 2014), or other forms of biochemically induced damage (Allan and Mitchell, 1977; Low et al., 1985; Nagababu and Rifkind, 2000; Cao et al., 2009), as noted above. This induces a bulging out and some curvature - also note that if there is a negative (i.e., separating) pressure this will also contribute to an initial curvature as sketched in the next upward figure - call this curvature $C_{0}$ as indicated in Figure 8Aii. We address this in more detail below.

The initial bleb, or “blister,” may propagate due to (i) a negative pressure induced by membrane/skeleton deformation as appears in our simulations (Zhu et al., 2017), (ii) additional binding of Hb or translocation of anionic lipids such as PS (Sheetz and Singer, 1974) leading to additional curvature caused fueled by biochemical energy, and (iii) the release of skeletal energy attached to the membrane following more intense skeletal deformation. Later we suggest a potentially important contribution arising from stored chemical energy associated with the asymmetry in lipid composition between the leaflets of the bilayer. Let us define ϵ_0_ as the elastic energy stored in the skeleton per unit area - this energy is released as the membrane separation spreads. This energy indeed depends on the deformation state as discussed in section 3.2. In a simple sense, the skeleton exerts a kind of “pinching action” on the membrane causing membrane/skeleton separation. The effect of skeletal deformation has been considered in several studies, e.g., those concerned with erythrocyte shape (Waugh, 1996; Lim et al., 2002; Mukhopadhyay et al., 2002) and vesiculation (Li and Lykotrafitis, 2015). In these citepd studies of minimum energy erythrocyte shapes (Lim et al., 2002; Mukhopadhyay et al., 2002) the same skeletal deformation that we consider here actually acts to inhibit budding, in their case to form echinocytes. In these studies skeletal energy was not estimated as we do by simulating actual cell deformations. This view is entirely consistent with ours, except that here we explicitly account for release of skeletal energy as membrane/skeleton separation occurs.

Note the geometric factors ℓ(*t*) and *h*(*t*), where ℓ is the current radius of the membrane separation and *h* is the height of the bulging membrane; here *t* may be viewed as a “time like parameter” marking the progression of the process (see Figure 8Aii). Vesiculation occurs soon after *h* → ℓ as discussed below. Note also, that there is the “work of adhesion” that must be done to de-bond the area of the membrane still attached to the skeleton; this called γ (per unit area); this has been discussed at length in the context of splenic vesiculation (Zhu et al., 2017). It is important to note that the membrane considered in Figure 8A is not open, i.e., finite, but is considered part of the overall cell's closed membrane. As budding occurs, additional membrane is drawn in as the vesicle's membrane area increases; this process requires the additional work of membrane/skeleton separation, γ, described below.

Now, for perspective, note that the bending energy of a spherical vesicle is simply 8πκ_*b*_, independent of its radius; κ_*b*_ is the membrane's bending modulus. On the other hand, the elastic energy stored in a circular membrane/skeleton patch of radius ℓ is $\pi \ell^{2}\epsilon_{0}$. This would seem to set a lower limit to the size of a vesicle that may form, i.e., we need

(1) $$\pi \ell^{2}\epsilon_{0}\geq 8\pi \kappa_{b}\;⤳\;\ell_{crit}\geq 2\sqrt{2}{\left\{\frac{\kappa_{b}}{\epsilon_{0}}\right\}}^{1/2}.$$

We next discuss the skeletal energy, i.e., ϵ_0_, and then the de-bonding energy γ.

### 3.2. Skeleton stored energy

**Figure 9:**
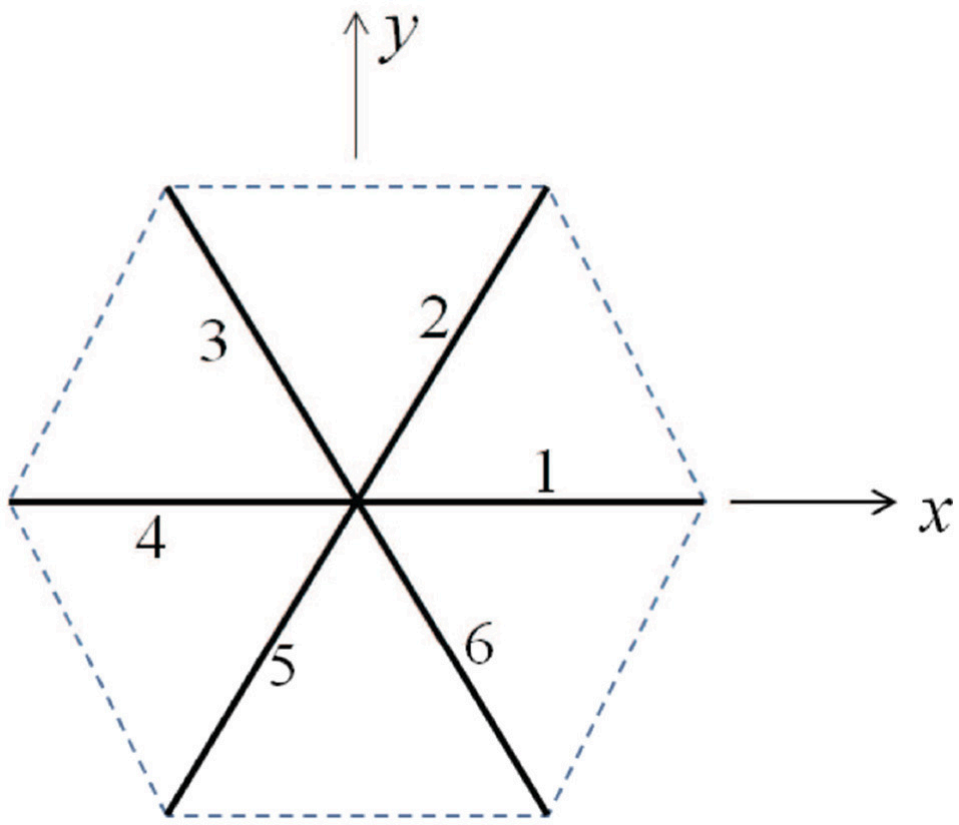
Simplified sketch of a junctional complex consisting of six Sps. The initial length of the JC spokes are taken as *L*_0_ = 40 nm here.

As shown in Figure 9, we consider a single JC consisting of six spectrin dimers (Sp), numbered 1 to 6. In its undeformed state the length of each Sp is _0_ so that the area covered by this hexagon is $A_{0}=\frac{3\sqrt{3}}{2}L_{0}^{2}$. We now consider a case in which the JC is stretched in *x* direction by a factor of λ_1_ and in *y* direction by a factor of λ_2_ (λ_1_ ≥ λ_2_), the corresponding area deformation is ψ = λ_1_λ_2_ and the shear deformation is χ = λ_1_/λ_2_. In the deformed state the lengths of the Sp become *L*_*i*_ (*i* = 1, ⋯ , 6).

Invoking the Worm Like Chain (WLC) model (Weiner, 1983; Zhu et al., 2007) the strain energy stored in the *i*-th Sp is Zhu et al. (2007)

(2) ϕi=kBTp(3Li2−2Li3/LcLc−Li),

where *k*_*B*_ is the Boltzmann constant. *T* is the temperature. *L*_*c*_ and *p* are the contour length and persistence length of Sp, respectively. In the following example we choose *L*_*c*_ = 144 nm, *L*_0_ = 40 nm, and *p* = 0.8 nm. The strain energy density is then calculated as $\overset{6}{\underset{i=1}{\sum }}\phi_{i}/\left(\psi A_{0}\right)$.

We note additionally that the erythrocyte skeleton is in a deformed state while attached to the cell's membrane “at rest,” i.e., without imposed deformations. This is revealed clearly in the observations of Vertessy and Steck (1989) and Shen et al. (1986) who observed skeletal shrinkage in Triton X-100 membranes. Such collapse of released membrane skeletons was reported by Levin and Korenstein (1991) and Truvia et al. (1998) in their studies of membrane fluctuations. In such studies skeletal contractions were at least on the order of a factor of 2.

### 3.3. Skeletal energy density vs. deformation modes, ϵ_0_

**Figure 10:**
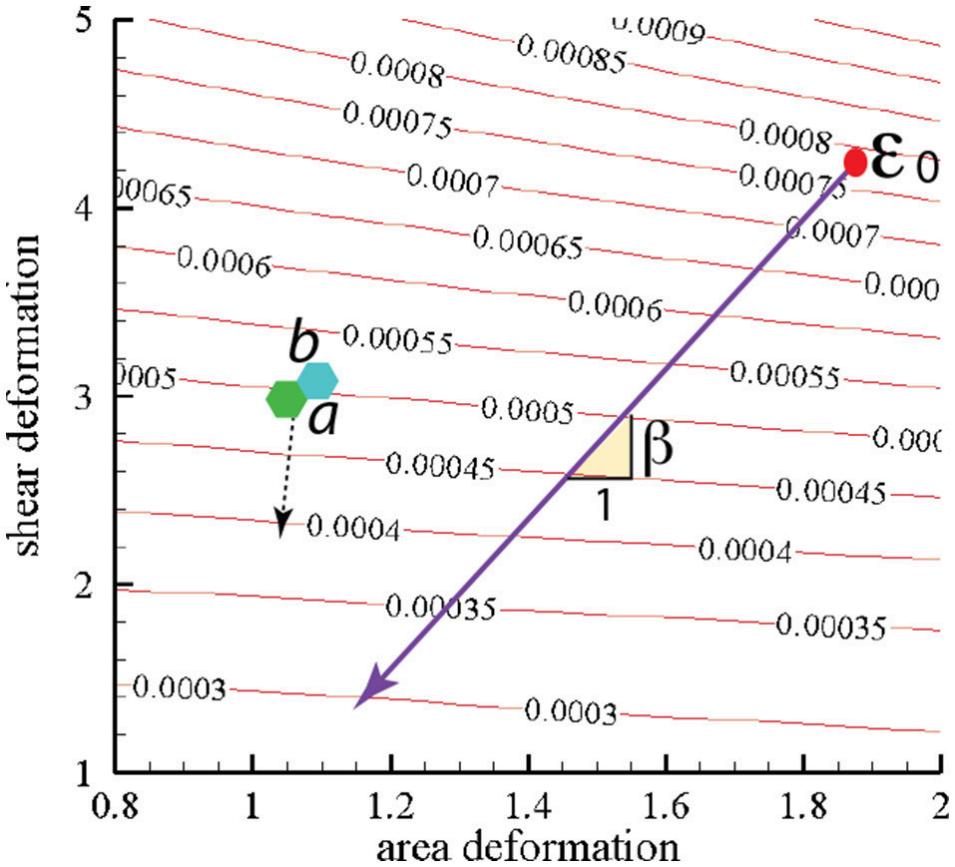
Stored skeletal energy density in units of Jm^−2^ vs. deformation mode. The heavy line indicates a possible path of reduced skeletal area and shear deformation. The points labeled “a” and “b” correspond to maximum energy densities achieved in simulations that are described in section 4. The broken line arrow suggests an energy path aiding promoting vesiculation.

In Figure 10 we present contours of ϵ_0_ as it depends of the deformation parameters χ = λ_1_/λ_2_ (representing shear deformation) and ψ = λ_1_λ_2_ (representing area deformation), where λ_1_ and λ_2_ are principal in-plane stretches and by definition λ_1_>λ_2_. We note that, within the range of deformations explored, we find $\epsilon_{0}\sim O\left(3-8\times 10^{-4}\right){\text{Jm}}^{-2}$.

We note this range would lead using Equation (1) to minimal separated membrane patch and hence vescile sizes in the range

(3) $$\begin{array}{l} 31.8\text{nm}≲\;\ell_{crit}≲51.6\text{nm}\\ \;63\text{nm}≲\;2\ell_{crit}≲106\text{nm} \end{array}$$

This is indeed a relevant, albeit lower, size range as noted, for example, by measured size ranges for vesciles formed during splenic flow (Bosman et al., 2012). It is also noteworthy that this areal size scale is comparable to that covered by a JC unit as depicted in Figure 9. This provides a basis for the comment made above in section 1.1 that vesicles with diameters less than, say, 40–50 μm are unlikely. For example, a membrane patch with a area of $\pi r_{\text{m}}^{2}$ would produce a vesicle of diameter *r*_v_ ~ 0.5*r*_m_ as follows from the membrane's area incompressibility, *i.e*. $\pi r_{\text{m}}^{2}=4\pi r_{\text{v}}^{2}$ for a spherical vesicle.

### 3.4. Work of membrane/skeleton adhesion, γ

Estimates for the work required to separate the membrane from the skeleton have been reviewed and analyzed recently by Zhu et al. (2017). Their analysis was based on experimental data measured for experiments on the formation of tethers. The results are somewhat tentative, yet would estimate γ to be of order $\gamma \sim O\left(1\times 10^{-4}\right){\text{Jm}}^{-2}$. This order is noteworthy when compared to our simulated range of the stored elastic energy in the skeleton, *viz*. $\epsilon_{0}\sim O\left(3-8\times 10^{-4}\right){\text{Jm}}^{-2}$. This implies that if (ϵ_0_ − γ) <0 this combination represents a driving force for vesiculation, although this assumes that as an area element of membrane/skeleton separates all of the stored elastic energy is released.

The heavy line in Figure 10 drawn at slope β represents a possible deformation path taken by the skeleton during its release from the membrane during a vesiculation process as discussed below.

### 3.5. Vesiculation simulations

Here we consider a simple scenario of spherical cap-like bud emerging from an initially flat membrane/skeleton section. The cap initiates from a circular patch, or raft, of radius *l*_0_ as shown in Figures 8(Aii,iii) and expands as a growing spherical cap with height *h*(*t*) and a fixed cord 2ℓ_0_. As the cap expands in area it draws in lipid bilayer and requires additional area of skeleton/membrane interface to separate.

Let the cap be of a sphere of radius *r*(*t*). The emerging cap's area is $A_{\text{cap}}\left(t\right)=\pi \left(h^{2}+\ell_{0}^{2}\right)$ and its radius is such that $2r\left(t\right)=\left(h^{2}+\ell_{0}^{2}\right)/h$; of course this means that as *h* → ∞, 2*r* → *h*. Also let ℓ be defined as the radius of a circular raft of membrane/skeleton with area of the spherical cap bud of Figures 8B,C. Hence the change in the area of skeleton/membrane area separated upon the cap's growth is $\delta A_{\text{ske}}=\delta \left(\pi \ell^{2}\right)$. But the change in the cap's area is $\delta A_{\text{cap}}=\delta \left\{\pi \left(h^{2}+\ell_{0}^{2}\right)\right\}$. Since the membrane is incompressible, $A_{\text{ske}}=A_{\text{cap}}$, and thus ℓ*δℓ* = *hδh* and ∂ℓ/∂*h* = *h*/ℓ.

Now the change in energy stored in the skeleton that is currently in the cap is

(4) ϵϵ=Δℰ=ϵ0{πl02π(h2+ℓ02)−1}πℓ2,

and this leads, upon differentiation with respect to *h* noting ∂ℓ/∂*h* = *h*/ℓ, to $\delta \epsilon^{\epsilon }=-2\pi h\epsilon_{0}\delta h$.

Next we note that as $\delta A_{ske}=2\pi h\delta h$ from above that the work expended in membrane/skeleton separation is δϵ^γ^ = 2π*γhδh*.

The energy of bending in the cap is given as

(5) $$\epsilon^{\kappa }=\frac{1}{2}\kappa_{b}\kappa^{2}A_{\text{cap}},$$

with κ taken as the mean curvature given by

(6) $$\kappa =\frac{4h}{h^{2}+\ell_{0}^{2}}.$$

With Equation (6) we have

(7) $$\epsilon^{\kappa }=8\pi \kappa_{b}\frac{h^{2}}{h^{2}+\ell_{0}^{2}}.$$

Note that as $h\to \infty ,\;\epsilon^{\kappa }=8\pi \kappa_{b}$ as expected. This yields upon differentiation

(8) $$\delta \epsilon^{\kappa }=16\pi \kappa_{b}\frac{h\ell_{0}^{2}}{{\left(h^{2}+\ell_{0}^{2}\right)}^{2}}\delta h.$$

Taken together we have

(9) $$\begin{array}{l} \delta \epsilon =\delta \epsilon^{\kappa }+\delta \epsilon^{\gamma }+\delta \epsilon^{\epsilon }\\ \;=16\pi \left\{\frac{h\kappa_{b}\ell_{0}^{2}}{{\left(h^{2}+\ell_{0}^{2}\right)}^{2}}-\frac{1}{8}h\zeta \right\}\delta h, \end{array}$$

with, as above, ζ = ϵ_0_−γ. To resolve this we may write *h* = αℓ_0_ and obtain

(10) $$\delta \epsilon =16\pi \left\{\frac{\alpha \kappa_{b}}{{\left(\alpha^{2}+1\right)}^{2}}-\frac{1}{8}\alpha \ell_{0}^{2}\zeta \right\}\delta \alpha .$$

Upon reintegrating Equation (10) we obtain

(11) $$\epsilon =8\pi \left\{\kappa_{b}\left[1-\frac{1}{1+\alpha^{2}}\right]-\frac{1}{8}\alpha^{2}\ell_{0}^{2}\zeta \right\}.$$

#### 3.5.1. Simulation results

Let us take $\zeta =\tilde{\zeta }\times 10^{-4}{\text{Jm}}^{-2}$, with $\tilde{\zeta }=1-4$. Also take $\kappa_{b}=\tilde{\kappa }\times 10^{-19}\text{J}$, with $\tilde{\kappa }=1-1.5$. Finally take $\ell_{0}=\tilde{\ell }\times 10^{-8}m$, with $\tilde{\ell }=1-4$; this puts ℓ_0_ in the range 10nm ≤ ℓ_0_ ≤ 40nm. With these definitions and values Equation (11) becomes

(12) $$\epsilon =8\times 10^{-19}\pi \left\{\tilde{\kappa }\left[1-\frac{1}{1+\alpha^{2}}\right]-\frac{1}{80}\alpha^{2}{\tilde{\ell }}^{2}\tilde{\zeta }\right\}.$$

### 3.6. Vesiculation: *pinching-off* as the final stage

The final stages of vesicle release may go as depicted in **Figure 12**. Here expansion continues as *h* increases along a downward slope of a ϵ *vs*. α(*h*) curve. At some stage the “bridge,” ℓ_0_, may itself retreat as the bud becomes more compliant with increasing radius, $r=\left(h^{2}+\ell_{0}^{2}\right)/2h$. As the angle θ defined in **Figure 12** decreases, the bending stresses in at the junction of the bud and bilayer main body increase and eventually rupture the bilayer. Spectrin may be retained within the vesicle, e.g., bound to Hb (Snyder et al., 1985), and/or released into the medium so as to remove skeletal proteins from the cell in rough proportion to lost membrane area in the vesicle as suggested by Ciana et al. (2017b). Resealing on the membrane is favored, of course, as is reattachment of the skeleton to the membrane.

**Figure 11:**
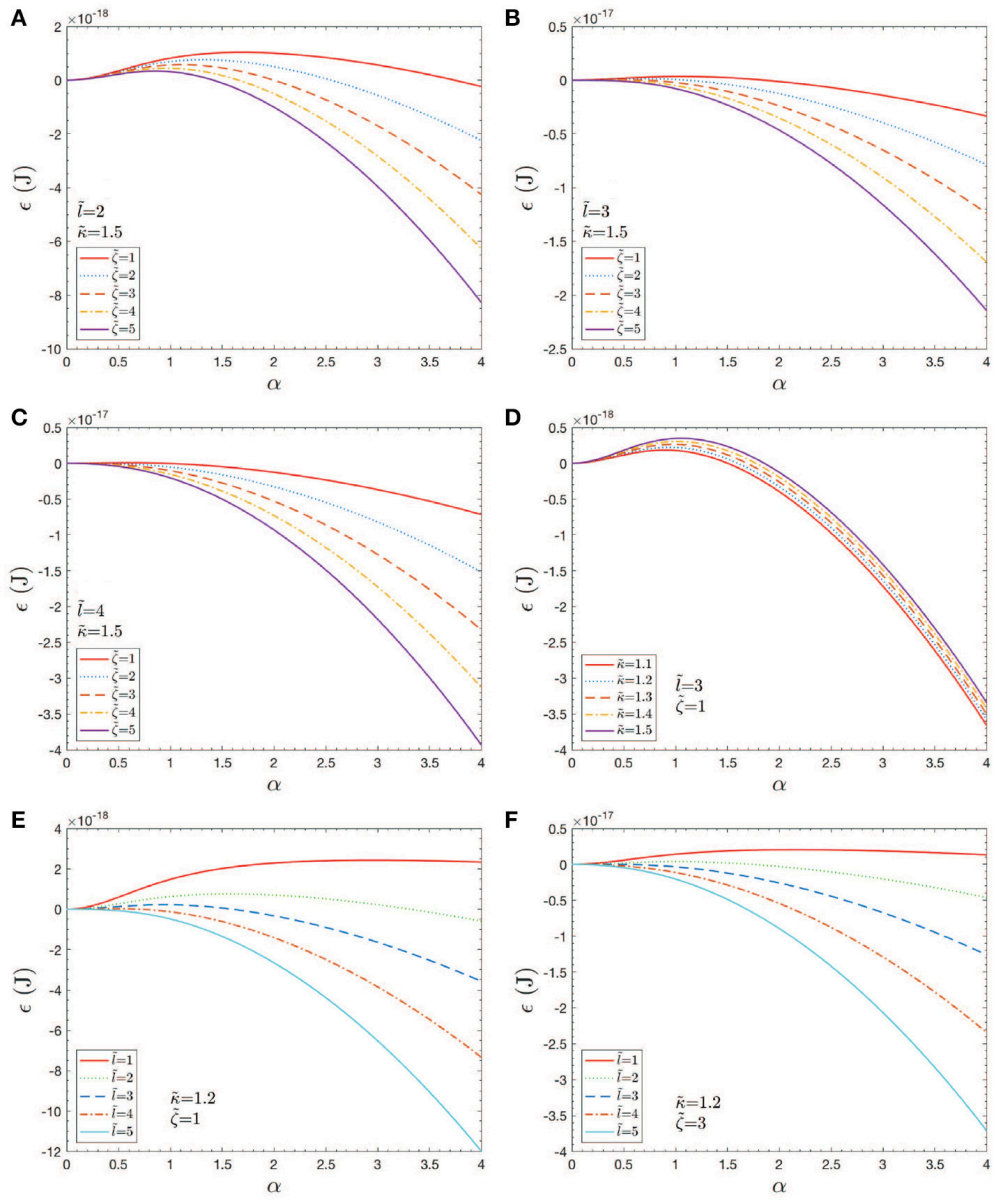
Case studies of total energy, ϵ vs. *h* = αℓ_0_. Note that an $\ell_{0}=40\text{nm }\left(\tilde{\ell }=4\right)$ would correspond to an initial skeletal/membrane separation covering about one JC unit. The parameters in the cases shown in **(A–F)** are listed within the figure.

**Figure 12:**
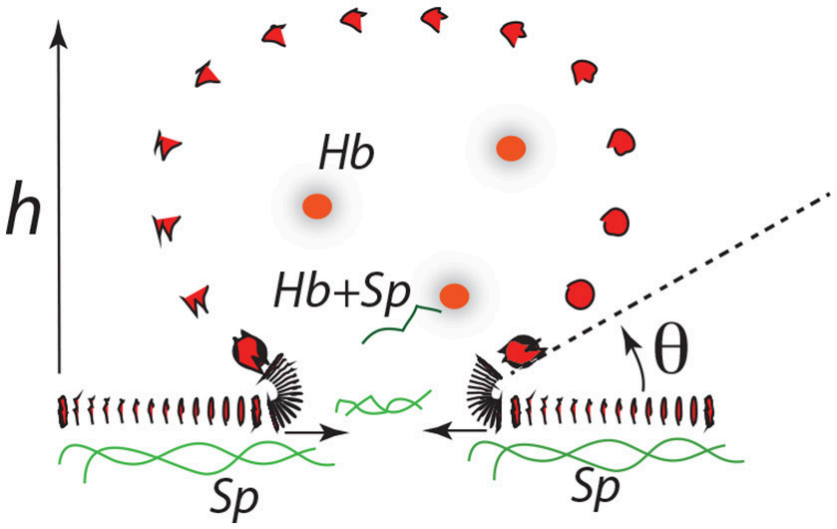
A budding vesicle of height *h* formed off an initial skeletal/membrane separation of radius ℓ_0_. The angle θ is the inclination of the vesicle membrane as it just leaves off the cell's membrane body. Note that as θ is reduced the curvature at that location increases dramatically.

As for estimates of vesicle size, we may proceed as follows. Consider as a first example Figure 11A; here the activation barrier is crossed at $\alpha \sim 1.2\left(\tilde{\zeta }=4\right)$ or $\alpha \sim 2\left(\tilde{\zeta }=1\right)$ with $\tilde{\ell }=2\text{ and }\tilde{\kappa }=1.5$. Now analysis of Figure 12 shows that

(13) $$\theta =\tan^{-1}\frac{2\alpha }{\alpha^{2}-1},$$

and hence if θ reduces so that θ → θ_crit_,

(14) $$\frac{2\alpha_{\text{crit}}}{\alpha_{\text{crit}}^{2}-1}\to \theta_{\text{crit}}.$$

Let us take, to explore the numerology, θ_*crit*_ = π/8; this yields α_*crit*_ ≈ 5. At this stage we would have $h_{v}\approx 5\times 2\times 10^{-8}\text{m}=100\text{nm}$. On the other hand if $\tilde{\ell }=4$, as in Figure 11C, we would find *h*_*v*_ ~ 200nm.

As a purely geometric criterion, Equation (14) would effectively define the length scale ℓ_0_ as the mediator of vesicle formation (at least vesicle size). Yet the vanishing of Δ*G*_*act*_ that depends also on κ_*b*_, ϵ_0_ and γ as well as on ℓ_0_ all play vital roles. However, it may be judged, that as long as Δ*G*_act_ → 0, ℓ_0_ may be judged as a (the) prime determinant of vesiculation. Of course, the very factors that influence γ as well as ϵ_0_, and possibly even κ_*b*_ are involved in causing an ℓ_0_ in the first place. With the numerology used here we find that predominantly vesicles are expected in the size range 100nm ≲ *h*_*v*_ ≲ 200nm; however, vesicles as small as *h*_*v*_~40−50nm are possible if sufficient driving energy, ϵ_0_ is available.

**Figure 13:**
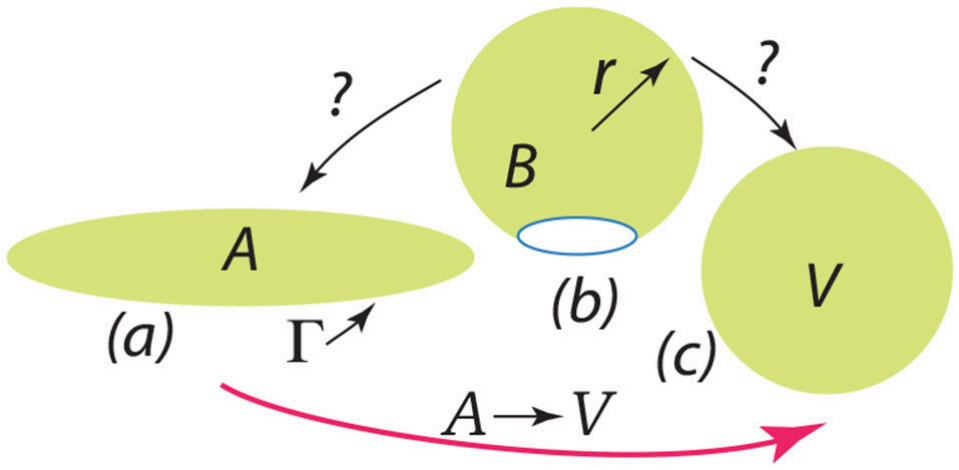
The depiction should be interpreted as follows: a bud (B) is released as in **(b)** and may revert to a flat patch (A) as in **(a)** or may close to form a spherical vesicle (V) as in **(c)**.

Finally we discuss the release of a severed bud, i.e., its closing to a spherical vesicle. The question is: once free, does the bud completely fold or revert to a flat disc with an exposed edge? This question is depicted in Figure 13. The severed bud (“B”) is depicted in Figure 13b and it may revert to a planar disc of area “A” as in Figure 13a or close to a vesicle (“V”) as in Figure 13c. The bud and the flat circular patch, however, possess an “edge,” Γ, with energy γ_*s*_ measured in units Jm^−1^ and which provides a driving force to close the bud or, if insufficient, fold a flat patch as in “A”.

The issue of the folding of an initially flat patch was considered by Helfrich (1974), and later by Fromherz (1983), and here we list a key result as phrased by Hu et al. (2012) who developed an approach based on these results for estimating values for the key parameters. In Equation 15 E is the energy along the path given by curve A→V in Figure 13.

(15) $$\begin{array}{l} \frac{ℰ(\xi ,\eta )}{4\pi (2\kappa_{b}+\kappa_{g})}=\tilde{ℰ}=\xi +\eta \{\sqrt{1-\xi }-1\},\text{ with}\\ \xi ={\left(\frac{ℛ}{r}\right)}^{2},\;\eta =\frac{\gamma_{s}ℛ}{2\kappa_{b}+\kappa_{g}},\text{ and }ℛ={\left(\frac{A}{4\pi }\right)}^{1/2}. \end{array}$$

Here ξ is a parameter that measures the progress along path A→B→V where ξ = 0 represents the initial flat patch of membrane of area A and ξ = 1 a full spherical vesicle of radius *R* (whose area is equal to A). The modulus κ_*b*_ has already been defined and κ_*g*_ is the so-called Gaussian modulus (Helfrich, 1974; Fromherz, 1983; Hu et al., 2012) (κ_*g*_ ≈ −κ_*b*_). Now as noted by Hu et al. (2012) - and is readily verified - the path A→B→V displays an activation barrier if η <2 at $\xi_{\text{act}}=1-{\left(\eta /2\right)}^{2}$; this amounts to $\Delta {\tilde{E}}_{\text{act}}={\left(1-\eta /2\right)}^{2}$. With this, our question is: as per Figure 13b, does the B revert to A or proceed to vesicle V after the driving energy supplied by, *inter alia*, ϵ_0_ and Δ*G*_*mix*_ has been expended?

To make use of these results we use representative values for κ_*b*_, κ_*g*_, and γ_*s*_ provided by Hu et al. (2012); in particular we take $\kappa_{\text{b}}\sim 10^{-19}\text{J},\kappa_{\text{g}}\sim -0.9\kappa_{\text{b}},\text{ and }\gamma_{\text{s}}\sim 3kT{\text{nm}}^{-1}\approx 1.2\times 10^{-11}{\text{Jm}}^{-1}$. Then with our defined geometry we find

(16) $$\begin{array}{l} ℛ={\left\{\frac{A}{4\pi }\right\}}^{1/2}=\frac{1}{2}\sqrt{\alpha^{2}+1}\ell_{0},\;\eta =0.55\tilde{\ell }\sqrt{\alpha^{2}+1},\text{ and}\\ \;\xi_{\text{act}}=1-0.076(\alpha^{2}+1){\tilde{\ell }}^{2},\text{ with }\ell_{0}=\tilde{\ell }\times 10^{-8}\text{m}. \end{array}$$

Now if at the point of bud severing α were in the range, say, α ≥ 2 and $\tilde{\ell }\geq 2$ we would judge that η≳2.6. In such cases no activation barrier would exist (from a flat disc) to form a closed vesicle. If, on the other hand, $\tilde{\ell }$ were modest in size, say $\tilde{\ell }\sim 1$ and α≲2 at budding then η≲1.22 and a barrier would exist to form a closed vesicle. In such cases one might suspect that severed bud would revert back to a flat disc, i.e., a “membrane fragment” as are often found.

## 4. Discussion of vesiculation simulation results of section 3

The results shown in Figures 11A–F provide an initial assessment of the key parameters, κ_*b*_, ζ = ϵ_0_−γ, and ℓ_0_. These are, in turn, affected by metabolic and biochemical influences as discussed above. Now given the magnitudes of the activation barrier, Δ*G*_act_ as indicated in Figure 11D, we expect vesiculation to essentially require Δ*G*_act_ → 0; we thereby take this as a possible criterion for vesicle release. For example, Figures 11A,D clearly indicate the effects of high values of κ_*b*_ and/or small initial membrane/skeleton separation ℓ_0_ in inhibiting vesiculation; the effect of ℓ_0_ is noteworthy. This perhaps obvious result is nontrivial as it suggests that the initial “triggering event” may have a mediating effect on the eventual size of the vesicle released, as opposed to the details of progression of skeleton/membrane separation and ongoing membrane bulging. Figures 11B,C,F support this view as they show that, even at modest-to-high values of κ_*b*_ and low values of ζ, Δ*G*_act_ may effectively vanish if ℓ_0_ is at least as large as a typical JC complex, viz. if ℓ_0_~40−50nm.

**Figure 14:**
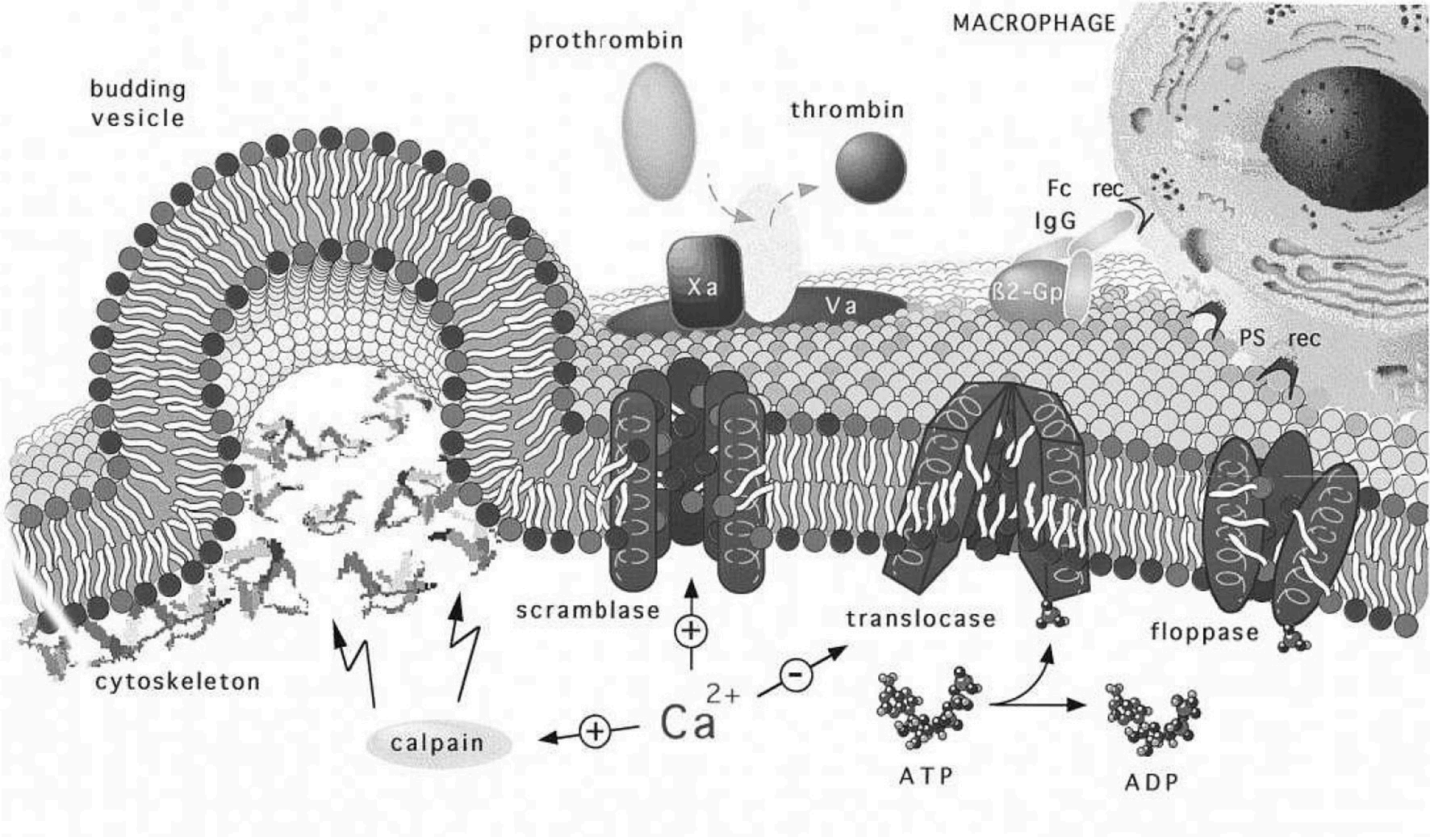
Regulation and physiology of membrane phospholipid asymmetry and vesiculation, taken from Zwaal et al. (1997). The effects of Ca^2+^ uptake on phospholipid randomization and on calpain activation that facilitates blebbing and release of PS exposing vesicles are addressed. We use the figure to also illustrate the geometry of a budding vesicle and to highlight how large curvatures develop and lead to pinching off of the vesicle bud. Note also that the large tensile strains in the membrane may increase the kinetics of randomization that initiates before vesiculation (Zwaal et al., 1997).

Next, we note that the magnitude of κ_*b*_ (Harmandaris and Deserno, 2000; Hu et al., 2012), the bending modulus, clearly plays a role in setting the height of Δ*G*_*act*_, and even determining if Δ*G*_*act*_ ≥ 0. In general, κ_*b*_ depends on membrane thickness and also on the bilayer membrane's lipid composition, including the lipid composition asymmetry. Here we introduce Figure 14, taken from Zwaal et al. (1997) that helps explain these issues. Yet, the prospect for vesiculation is also determined by the relative magnitudes of the terms ϵ_0_ and γ given in Equation (11) and (12) via ζ = ϵ_0_−γ. We continue to discuss the prospects for Δ*G*_act_ → 0 below. For now we note that Equation (12) reveals an activation barrier at $\alpha^{2}=\sqrt{80/\mu }-1$ with $\mu ={\tilde{\ell }}^{2}\tilde{\zeta }/\tilde{\kappa }$. This shows, after substitution into Equation (12), that if μ/80 <1 an activation barrier exists with Δ*G*_*act*_>0; conversely, we have that

(17) $$\mu /80={\tilde{\ell }}^{2}\tilde{\zeta }/\tilde{\kappa }\geq 1$$

implies that, once initiated, a bud will spontaneously grow.

Now the roles of calpain and Ca^2+^ uptake in vesiculation have been already discussed and are referred to in Figure 14. We take the depiction of budding as shown there to represent a stage in any of our Figure 11 but along a downward slope of a ϵ *vs*. α(*h*) curve; note the curvatures at such stages, most particularly at the sites where the bud meets the membrane body. As such stages we envision that phospholipid asymmetry has been lost (or partially lost) and that the membrane is under considerable tension at what is to be the “pinching off” sites located as just referred to above. This is addressed in more detail below as we suggest how vesiculation is completed, but after a relevant diversion concerning the spectrin skeleton.

### 4.1. Disposition of skeletal proteins during vesiculation

A question arises suggested by the Zwaal-Schroit depiction of Figure 14 as well as by Figure 12 below, viz. *what happens to the spectrin contained in the released skeleton?* It has been reported that shed vesicles (MV's) are deficient in skeletal proteins, viz. spectrin (Lutz et al., 1977; Dumaswala and Greenwalt, 1984; Knowles et al., 1997; Willekens et al., 2003a,b; Bosman et al., 2012) and this has suggested that spectrin may remain in the cells. Ciana et al. (2017a,b) have, however, recently shown that the cells that shed vesicles, *in vivo*, lose spectrin in rough proportion to their membrane area; no data was presented on the spectrin content of the shed vesicles, however. The fact is that the number of vesicles found in venous blood is orders of magnitude too low to account for the cell membrane lost. This means that vesicle membrane is recycled without entering general circulation, i.e., presumably within the organ where the vesicle forms. Yet Snyder (Snyder et al., 1985) had already shown that vesicles shed from “young cells” (i.e., cells so classified by their low density) contained “Hb-spectrin complexes” suggesting that spectrin was also shed into vesicles in at least some amounts. Hence we speculate that spectrin may indeed be lost to either the medium and/or retained within shed vesicles along with Hb as is repeatedly reported. In fact, examining either Figures 14, 12 or Figure 2 of Alaarg et al. (2013) suggests that, since the pinching off process produces very large local deformations, the skeleton would bed severed and retained within the vesicle or the medium of the organ producing vesicle. As many reports of spectrin deficient vesicles are those produced *in-vitro* we discuss this further and will suggest, in the Discussion, that our oscillatory shear methods be utilized to study the character of vesicles produced via our splenic-like deformations. This logically leads us to more detailed consideration of γ and ϵ_0_.

### 4.2. Biochemical energetics

As noted in section 3 most prominently, and elsewhere, attachment between the skeleton and membrane can be reduced by a number of biochemical and metabolic causes. Hence we realize that although imposed deformations that increase ϵ_0_ will stimulate vesiculation, e.g., during splenic flow or under our conditions of oscillatory shear flow, even modest states of deformation are sufficient to induce the process. An intriguing possibility is that the free energy released by losing phospholipid concentration asymmetry may provide some of the required energy associated with ϵ_0_. This remains to be explored, but here we make a simple estimate of such a contribution to test the idea's viability. The fact is that significant metabolic free energy is expended in creating lipid concentration asymmetry (Zwaal et al., 1997; Fadeel and Xue, 2009; Bevers and Williamson, 2016) and we wonder what portion of it may be available to perform the work of vesiculation.

We take a simple view and consider the primary lipids that “mix” in a final state of symmetry from an initial state of asymmetry between the outer and inner bilayer leaflets to be grouped into 2 groups. Let group #1 consist of sphingomyelin and phosphstidylcholine (initially located on the outer leaflet) and group #2 contain phosphatidylethanolamine, phosphatidylserine, and phosphatidylnositol (initially on the inner leaflet). We will call the molecular fraction of either group “*x*” since we assume a perfect solution within the leaflets.

We use the well known relation for the entropy of mixing for a perfect solution, viz.

(18) $$\Delta g_{mix}=-nk\{x\ln x+(1-x)\ln(1-x)\},$$

where, in context, *n* is the total number of lipids in a leaflet, *k* is Boltzman's constant, and *x* is either group #1's or #2's molecular fraction. We use Equation (18) to compute

(19) $$\Delta S_{mix}=\Delta g_{\text{sym}}-\Delta g_{\text{asym}},$$

and note that the free energy change, just associated with perfect mixing, is Δ*G*_*a*→*s*_ = −*TΔS*_*mix*_; we will take *T* = 300 K. We take for *n*, the number of lipids per leaflet, 1.4 × 10^18^*m*^−2^ ≤ *n* ≤ 1.5 × 10^18^*m*^−2^ (White and King, 1985; Giang and Schick, 2014) (this being computed from the average area per lipid, *a*~0.68−0.70nm^2^) and *kT*~4 × 10^−21^*J*. For × we take *x*~0.74 (Zwaal et al., 1997; Fadeel and Xue, 2009). These numbers yield $\Delta G_{\text{mix}}\sim -\left(6.7-7.2\right)\times 10^{-4}{\text{Jm}}^{-2}$, which is in the range of ϵ_0_, a primary driving force for vesiculation (see Figure 10). Even if a portion of this stored free energy were made available it could drive vesiculation. For perspective, we recall that the standard free energy of hydrolysis of ATP is in the range $\Delta G_{\text{ATP}}^{{}^{\circ}}\sim -\left(11-13\right)\text{kcal/mole}\sim -\left(7.5-9\right)\times 10^{-20}\text{J/ATP}$ (Alberts et al., 2002). Hence one ATP per 10^2^−10^3^ lipids could fuel vesiculation. We note Beleznay et al. (1994) and Zachowski (1993) report that transport of lipids such as PS consumes about one ATP per lipid, i.e., an order of magnitude larger than we estimate for Δ*G*_mix_ per lipid - as such lipid transport must have a substantial activation barrier, this seems quite reasonable. *Interactions and association of PS with spectrin have been documented* (An et al., 2004a,b; Grzybek et al., 2006) *and those may be disrupted during membrane/skeletal separation, promoting translocation*.

Thus for the above reasons we expect reductions in γ and increases in ℓ_0_ are prime contributors to, and even mediators of, vesiculation. A simple view would be that the skeletal energy density, ϵ_0_, would be augmented by −Δ*G*_*a*→*s*_ as a prime driver of vesiculation. As imposed deformations, i.e., ϵ_0_, have the prospect of promoting the process as we explore next regarding imposed oscillatory shear flows in the specific context of the above vesiculation model prospects. Finally we address the “pinching off” process that releases the vesicle.

### 4.3. Pre-curvature and biochemically induced curvature

Returning to Figure 11, we recognize that when Δ*G*_act_>0 we are typically in scenarios where α ≲ 1−1.5 yet if we began the process at, say α≳1.5 in most cases Δ*G*_*act*_ would vanish. We thereby ask about the effects of pre-curvature, or spontaneous curvature, as may arise, for example, by the expected trends to randomize the lipid concentration between the inner and outer leaflets (Zwaal et al., 1997). As noted Sheetz and Singer (1974) anionic molecules such as PS that are known to partition to the outer leaflet with vesiculation will indeed tend to cause crenation, i.e., outward budding (see also Deuticke, 1968). Just above we have shown that the thermodynamic driving force for this can be, indeed, substantial. A simple analysis of such induced curvature might go as follows. Simply consider Equation (7), the bending energy, and equate it to a portion of the free energy of mixing Δ*G*_mix_; call this portion $\Delta {\tilde{G}}_{\text{mix}}$. This yields

(20) $$8\pi \kappa_{b}\frac{h^{2}}{h^{2}+\ell_{0}^{2}}=-\Delta {\tilde{G}}_{\text{mix}},$$

or

(21) $$|\Delta {\tilde{G}}_{mix}|=8\frac{\alpha^{2}}{{\left(\alpha^{2}+1\right)}^{2}}\frac{\kappa_{b}}{\ell_{0}^{2}}.$$

For some numerology, take α = 1, $\kappa_{b}=10^{-19}$J and recall the definition of $\tilde{\ell }$ to obtain

(22) $$|\Delta {\tilde{G}}_{mix}|=2\frac{10^{-3}}{{\tilde{\ell }}^{2}}=\{\begin{array}{l} 5\times 10^{-4},\;\tilde{\ell }=2\\ 2.2\times 10^{-4},\;\tilde{\ell }=3\\ 1.25\times 10^{-4},\;\tilde{\ell }=4,\text{ etc}. \end{array}$$

Hence we may envision that in all the cases shown in Figure 11, taken as examples, Δ*G*_*act*_ may be driven to near zero. Now the reader will notice that as we take ℓ_0_ larger and larger, the required $\Delta {\tilde{G}}_{mix}$ reduces, eventually as $\Delta {\tilde{G}}_{mix}\sim 1/\alpha^{2}$.

These arguments also demonstrate that the effects of the cell membrane's natural curvature are quite modest due to the much larger curvatures involved in creating a budding vesicle; that is, the cell's surface appears to be relatively flat compared to the emerging vesicle.

### 4.4. Vesiculation in oscillatory flow: final analysis

Taken together, the results herein and those of sections 2 and 3 would support a scenario such as this: imagine a deformation state such as represented by points “a” or “b” of Figure 10 that can arise from the simulations we described. Now imagine an augmentation of the associated $\epsilon_{0}\sim 5\times 10^{-4}{\text{Jm}}^{-2}$ of order, say $-\Delta G_{a\to s}\sim O\left(2-3\times 10^{-4}{\text{Jm}}^{-2}\right)$. This places the total prime vesiculation driving force at ~7−8 × 10^−2^Jm^−2^, that according to the result scenario's of Figure 11 could very probably produce vesiculation.

A further assessment of vesiculation prospects with the examples of Figures 4–6 could go as follows. Consider the vesiculation simulations as, say, in Figure 11. From Figure 11C, for example, looking at the contours of energy density we would conclude that if there were regions—call them *aged regions*—where skeletal/membrane disruptions had occurred over areas of, say, $\tilde{\ell }\geq 3$ the probability of vesiculation at regions where $\tilde{\zeta }\geq 2$ would be quite high; at regions where $\tilde{\zeta }\geq 4$ vesiculation would be judged to be nearly assured. This follows since in this case Δ*G*_*act*_ → 0. Hence, in Figure 6 we would conclude that at any region displaying a red color we expect vesiculation to occur in cells containing aged areas where $\tilde{\ell }≳3-4$. In Figure 4 which also displays deformation states quite similar to those developed in splenic flow (Zhu et al., 2017) we would likewise focus on those regions in the lower-right foreground. Note, here we take γ = 1 × 10^−4^Jm^−2^ (Zhu et al., 2017) and that the red areas display $\tilde{\zeta }\geq 3$. On the other hand, the case shown in Figure 3B displays somewhat reduced levels of stored energy, i.e., $\tilde{\zeta }≲3$ and hence we judge that vesiculation may require larger aged areas involving, say $\tilde{\ell }≳4-5$.

Similar case studies can be made and from such we could well conclude that during splenic flow — as closely replicated by our oscillatory flow - vesiculation is highly probable providing aged regions exist covering nearly the area of a single JC unit (i.e., corresponding to $\tilde{\ell }\sim 3-4$). This would surely classify the spleen as an effective filter as expected — it might also support the notion that the majority of such *self-protection vesicles* are produced *in vivo* in such flow conditions, e.g., in the spleen and elsewhere where such deformations are imposed.

### 4.5. Shear induced erythrocyte membrane trauma

Given the flexibility and inherent time and rate dependence of RBC deformation, it is unsurprising that cell damage induced by imposed shear displays a complex phenomenology. For perspective *vis-*à*-vis* oscillatory shear, as we have presented it, we briefly review some background. We begin by noting that the physiological range of shear stress imposed upon the RBC is often put in the range of $\bar{\tau }\sim 5-10\text{Pa}$ (Meram et al., 2013); it is hence noteworthy that our forecasted average shear stresses presented herein for oscillatory flow are in that range. However, as explained early on by Leverett et al. (1972), the question of membrane rupture depends not only on stress level but also on the time of exposure suggesting perhaps “creep-like” damage mechanisms are operative. Moreover, stressing rate is important (Li et al., 2013) and in the case of cyclically imposed stresses, both frequency and amplitude are important (Watanabe et al., 2006; Wantanbe et al., 2007; Hashimoto, 2014; McNamee et al., 2016); this suggests that even wave form for periodic stressing would likely be still another factor. In fact, patterns using stress (flow) bursts coupled to pauses (i.e., hold times) have been employed (McNamee et al., 2016; Horobib et al., 2017). Nevertheless, when stresses are imposed for long times (≳60 min) a critical shear stress to induce cell lysis in the range τ_crit_~150*Pa* has been quoted (Leverett et al., 1972); other reports place this higher at τ_crit_~250*Pa* (Sutera and Mehrjardi, 1975). Yet—and this is important!—in a study involving exposure to shear stresses τ~10Pa, but for 2 h, it was found that such exposed cells were recognized and sequestered by rabbit spleens (Sandza et al., 1974). That is, such cells were sequestered and removed during splenic flow. Here we may speculate that although such exposure was *sublethal* (i.e., did not cause bursting, cell distortion, or other residual damage), it did induce *cell removal processes*, perhaps vesiculation. Note that the time to induce possible vesiculation in Sandza et al. (1974) was unknown, but the stress level at τ~10Pa is just at the levels we forecast for oscillatory flow as in the case of Figure 5. Finally, we note that human splenic flow passage times are <1*s* (MacDonald et al., 1987; Zhu et al., 2017). Given that multiple passages may be involved, we take the stress protocol to involve multiple passes with durations of, say 0.02-1 s; these to employ shear stress levels of τ~5−10Pa. In oscillatory flow we would suggest frequencies in the range ν~1−50Hz.

We also note that our simulations methods can be used to analyze the deformations experienced by RBC's subject to all the flow patterns imposed in the studies cited above, and can thereby be used to assess potential damage, lethal or sublethal, that may be caused. Moreover, it is possible to include interactions between cells and/or cells and structural features such a channel walls so that more quantitative assessments can be made of possible influences they may have on cell response.

## 5. Supporting experimental evidence

To confirm the effects of oscillatory flow as we have described them and to assure that our proposed imposed deformations do not cause lethal damage to a significant population of cells we used flow cytometry to explore the process of vesiculation and possible fragmentation following typical programs of shear. We imposed both modest and somewhat severe deformations to more completely explore the extent of possible cell damage via fragmentation and vesiculation. We suggest below, however, that in exploring aging induced vesiculation only the more modest deformations we have described be imposed. For example, an oscillatory frequency of 10 Hz appears to be reasonable since it coincides with the typical duration (0.1 sec) of slit passage in splenic flows (MacDonald et al., 1987). The shear stress on the cell depends on both the applied shear rate and the viscosity of the surrounding fluid. To match the typical shear deformation of the skeleton in splenic flow (the area deformation is negligibly small) this stress should be 5–10 Pa as described in section 4.5 and by the various plots of deformation in oscillatory flow we have included.

### 5.1. Materials and methods

#### 5.1.1. Blood samples and reagents

Adsol (Fenwal Laboratories, Deerfield, IL) preserved and leukoreduced red cell units were purchased from the San Diego Blood Bank (San Diego, CAI). Glycophorin A-PE, Annexin V FITC, and annexin V-binding buffer concentrate were purchased from BD Pharmingen (San Jose, CA). Flow Cytometry Absolute Counting Standard microbeads (6–7 μm) were purchased from Bangs Laboratories, Inc. (Fishers, IN). Megamix, a blend of monodisperse fluorescent beads of three diameters (0.5, 0.9, and 3 μm) was purchased from BioCytex (American Diagnostic, Hauppauge, NY, United States).

#### 5.1.2. Oscillatory shear

The steady and oscillatory shear conditions were applied on a stress-controlled shear rheometer (T.A. Instruments, model AR-G2), with a cone-plate of 60 mm diameter and 1° of angle, and with a plate-plate geometry of 60 mm diameter and a gap of 100 μm. The shear flows are imposed with a duration of 1–2 s, and repeated for multiple cycles, and carried out at 37°C.

#### 5.1.3. Isolation and purification of red cell vesicles from shear RBC suspension

Isolation of vesicles from the RBCs suspensions were completed by high-speed centrifugation as previously described (Kriebardis et al., 2008). Briefly, 1-mL aliquots were removed and centrifuged at 2,000 g at 4°C. The supernatant was centrifuged once again to ensure the absence of any RBCs and immediately filtered through sterile 0.8 mm pore size syringe-driven nitrocellulose filter units (Millipore, CA, United States). The supernatant was ultra-centrifuged at 37,000 g at 4°C for 1 hour, and the pellet of vesicles was resuspended in PBS and ultra-centrifuged twice under the same conditions.

#### 5.1.4. Quantitation of micro-vesicles using flow cytometry

**Figure 15:**
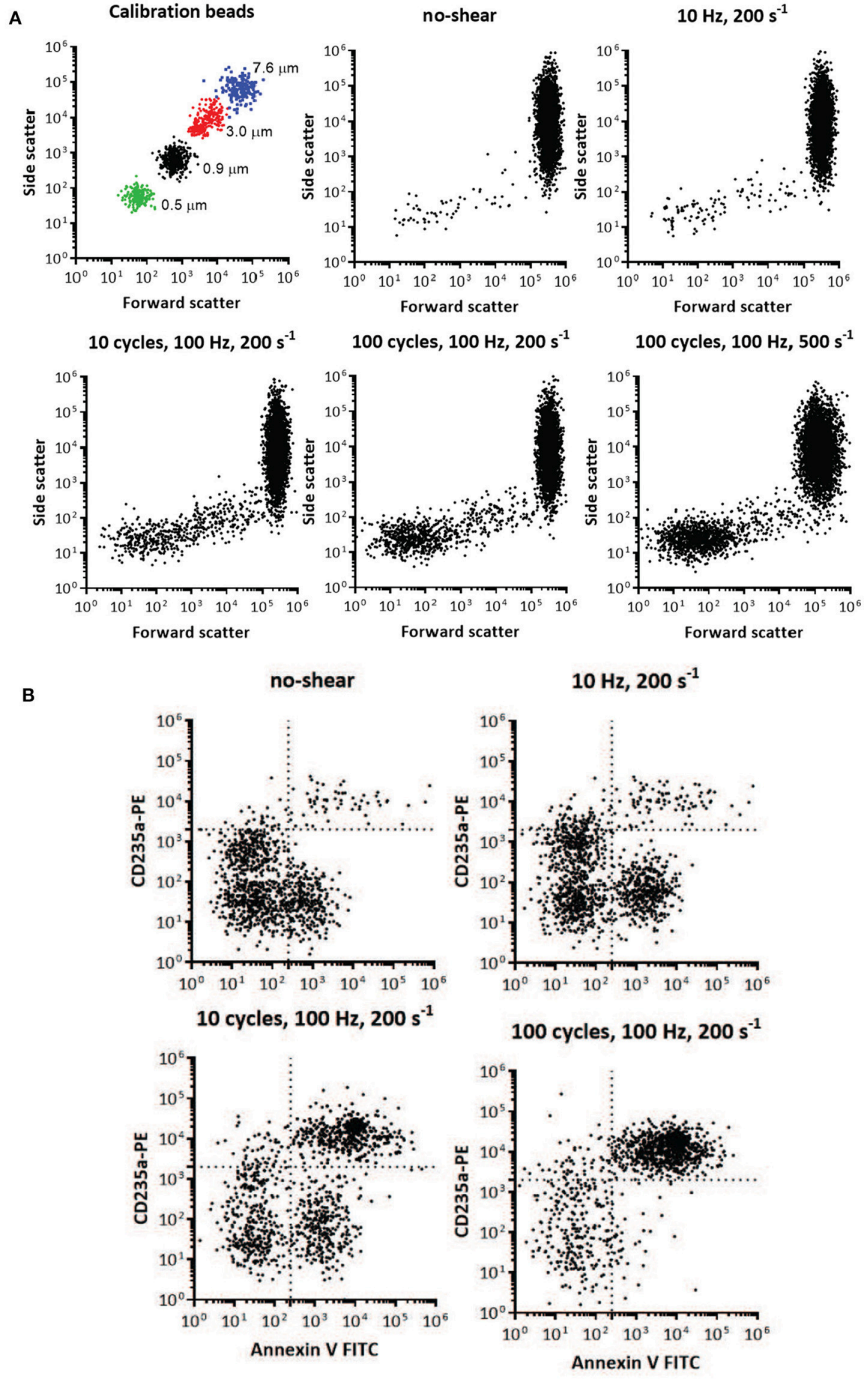
Flow cytometry identification of presence and size of RBC vesicles based on the light scatter parameters and quantification of RBC vesicles based on marker for phospholipid PS and glycophorin A (CD235A) using flow cytometry. **(A)** Light scatter results using side scatter (SSC) as the indicator of shape and forward scatter (FSC) the indicator of size. Top left panel shows light scatter parameters of the mixture of standard calibration beads (7.6, 3, 0.9, and 0.5 μm). Other panels show light scatter parameters of the blood samples before mechanical shear (no-shear), and after being subjected to different frequencies, cycles, and degrees of oscillatory shear. Inspection of the scatter from the calibrated beads and the blood subjected to mechanical shear indicates the increasing presence of vesicles below 1 μm in size. Direct size comparison cannot be made between beads and vesicles, since beads have a higher index of refraction, and therefore lower size threshold, than vesicles (Lacroix et al., 2010; Yuana et al., 2011). **(B)** Flow cytometry analysis of RBC vesicles extracted from blood before oscillatory shear (no-shear), and after being subjected to different frequencies and number of cycles of oscillatory shear. Only events positive to anti-CD235a and Annexin V were defined as RBC vesicles (Kriebardis et al., 2008). The number of calibration beads was counted to determine the absolute number of RBC vesicles. The number of cells was approximately 10^7^.

Red cell vesicle analysis and enumeration was obtained using a FACS Aria flow cytometer (BD Biosciences, San Jose, CA, United States). To confirm the formation of vesicles after imposed shear, the side scatter and forward scatter events from size calibration beads (0.5, 0.9, 3, and 7.6μm,) was compared to the side scatter and forward scatter events of a small volume of supernatant and erythrocytes of blood centrifugated at 2,000 g at 4°C (Figure 15A). These results were used to determine the resolution of the instrument, and to confirm the presence of vesicles and smaller cell fragments as the amount of imposed shear deformation increased (Figure 15A). The size range was confirmed and corrected by spiking a blood sample with calibration beads to establish the effect of the calibration beads' higher index of refraction on size relative to membrane vesicles (lower size threshold than membrane vesicles) (Yuana et al., 2011). Purified vesicles from RBC suspensions were labeled with glycophorin A-PE and annexin V FITC at ice temperature and protected from light. Red cell vesicles were discriminated by size and further defined as CD235a-PE and Annexin V positive event. Vesicle counts were calculated by adding a predetermined number of calibration beads to each blood sample, and from the nominal number of beads added per volume of sample a total number of vesicles was calculated.

### 5.2. Results

Two-color flow cytometric analysis, employing a combination of antiglycophorin A and Annexin-V-FITC, has demonstrated that shear deformations induce vesiculation as theoretically forecasted herein. Flow cytometry results are shown in Figure 15, where size is one of the key factors defining these events. Flow cytometry allowed us to obtain information on the morphology (size and granularity) of vesicles formed due to imposed shear, as evidenced by the forward scatter and side scatter of vesicles compared to calibration beads of appropriate diameters (Figure 15A). Forward scatter properties were used as an estimate of size, and side scatter properties were used as an estimate of particle shape. Forward and side scatter events from size calibration beads were used to resolve the instrument sensitivity and detection range. Figure 15A shows that the sub-population of vesicles increased with shear deformation as forecasted. Examination of the top-left scatter plot in Figure 15A indicates that 0.2 μm is the lower limit of detection for the beads. Vesicles are defined as membranous vesicles of arbitrary size from 0.2 to 1.0 μm. In contrast to RBCs, vesicles derived from RBCs may expose negatively charged phospholipids toward their surface. Living cells, however, employ energy-dependent mechanisms to actively shift negatively charged phospholipids to the inner membrane leaflet as discussed in section 4.2. Flow cytometric observation allows for the determination of the fraction of vesicles that bind annexin V, which suggests exposure of negatively charged phospholipids. Figure 15B shows that the fractions of glycophorin A positive and exposed PS vesicles (observations in the upper right quadrant) increased with shear deformation. Fluorescence events from antiglycophorin A and Annexin-V-FITC showed that the erythrocyte vesicles that expose PS, also had a high expression of glycophorin A.

Moreover, these results prove that oscillatory shear flows do not damage a significant portion of the RBC's. As an example, the results in Figure 15 indicate that after a protocol of 100 cycles, of shear rate σ = 200*s*^−1^, at 100 Hz we found the number of vesicles to be $O\left(10^{4}\right)$. As this protocol corresponds, based on the deformations experienced by the cells, to approximately 10,000 *in-vivo* splenic slit passages for each cell we would expect that given our 10^7^ cells this would have resulted in a number of vesicles of $O\left(4.8-6.5\times 10^{9}\right)$ *in-vivo*. In this we assumed that *in-vivo* a vesicle is produced at a rate of approximately 2–2.71 per day per cell (Willekens et al., 2003b; Ciana et al., 2017b). Hence as this expected *in-vivo* vesicle count is clearly much larger than our result of $O\left(10^{4}\right)$ we conclude our methods do not induce spurious cell damage or unexpected vesiculation. In terms of *in-vivo* splenic passages the deformation histories experienced by cells in this protocol would have occurred over about 250 days (if cell deformations outside of the spleen are not considered), or more than twice of a cell's average lifetime. Thus, a reason for the difference is that cells would not be expected to metabolically age during as they would *in-vivo* in the quite short durations of our tests. Additionally, we imposed somewhat more severe deformations (shear rate amplitude 2,000 s^−1^ at 50 Hz and with 100 cycles with durations of 2 s) to further explore the extent of possible cell damage. The basic hemologic parameters demonstrate that the majority of the cells (> 95%) survive the treatment.

Vesicle counts found per microliter of plasma were as follows: 217 for our control with no-shear and 986, 23,034, and 46,026 for the cases of 1 cycle at 10 Hz, 10 cycles at 100 Hz and 100 cycles at 100 Hz, all with a shear rate of σ = 200s^−1^, respectively. The number of 217 vesicles per μl of plasma is consistent with and helps confirm previous such findings, e.g., those of Willekens et al. (2003b) who reported the range 61-308 with an average of 169 vesicles per μl of plasma.

Measurements have shown that the volume rate of blood flowing through 100gms of spleen is approximately 170 ml/min/100 gms (Oguro et al., 1993). Assuming the spleen weighs, on average, 150gms and the body contains 4.5l of blood we estimate that the splenic passage rate of a typical erythrocyte is about 80 per day. However, not all these passages involve passages through the venous slits of the red pulp; in fact depending on mammalian species and degree of health versus diseased states, up to 90% of the blood flow may bypass the filtration beds of the red pulp (Schmidt et al., 1993; Cesta, 2006). Thus, the precise rate of erythrocyte passage through the venous slits is difficult to specify and here we make the assumption of 50%. Hence we arrive at an estimate for, discussion sake, of ~40 slit transits per cell per day. We thereby envision they undergo a pulsed-like deformation with a frequency of say $\nu_{f}\sim O\left(0.0005\right)\text{Hz}$. Hence we suggest that our lower frequency test protocols are more appropriate for studies of age induced vesiculation. Now the most severe deformation occurs within a time scale of 0.1 s. A realistic deformation protocol would thus be to subject cells to one oscillation cycle, pause for 2160s, start a second cycle, and repeat that for ~40 × 120~4, 800 times (as a cell does in typical circulation). As this may not be practical, we believe the approach we use, i.e., subject a population of cells to high frequency oscillatory flow for 1–2 s and repeat for O(10-100) cycles is an attractive, viable alternative; this is consistent with physiological deformation time scales as discussed in section 4.5. Indeed, we also suggest that lower shear rates such as σ = 200−500s^−1^ be explored as we have done.

## 6. Concluding discussion

To illustrate the versatility of our proposed methods, we revisit the discussion of section 4.1 concerning the disposition of skeletal proteins, viz. spectrin, during vesiculation. As noted there, Ciana et al. (2017b,a) have shown that cells that shed vesicles *in-vivo* lose spectrin in proportion to their loss of membrane; this makes sense given the cell's need to maintain a functional mechanical membrane/skeleton structure. Now consider the *in-vitro* experimental findings of e.g., Knowles et al. (1997) who induced vesiculation via micro-pipette aspiration. Indeed, (i) skeleton/membrane separation, (ii) membrane vesicle ejection, and (iii) skeleton retraction back into the cell, were all observed. Hence, these vesicles were indeed deficient in spectrin and we speculate also in anchoring proteins. This process was successfully simulated and explained by simulation with an earlier version of our computational models (Peng et al., 2010). In short, what we found in Peng et al. (2010) was that the deformations and their time scales cause very significant skeletal restructuring at regions of eventual vesiculation. This was due to the viscous drag of anchoring proteins (viz. band 3 and glycophorin C/JC) and results in very large (~ 80–90%) reductions in areal densities of skeleton anchoring sites. This, combined with the large disassociation stresses at the aspiration's tip, causes the membrane to “tear away,” or rather “pull away,” from the cell and skeleton where the latter retracts back into the cell. But we later showed that such a scenario does not occur during splenic flow due to the very different time scales and hence reductions in anchoring density must occur otherwise (Zhu et al., 2017) . That is, during *in-vivo* splenic flow, there is not time for mechanically driven reductions in anchoring density. This explains the important role our analysis parameter ℓ_0_ plays in deciding the progress of vesiculation. Indeed, if ℓ_0_ were very large, say ℓ_0_≿40−80nm, as it may well be in *in-vitro* experiments, vesiculation would readily occur via a different pathway as suggested by Ciana et al. (2017b,a). Since our methods are designed to reproduce both the deformation histories, with the appropriate time scales, as occur in splenic flow, we suggest that the character of vesicles produced in oscillatory flow be examined with various degrees of membrane/skeleton disruption.

We add that as far as the effects of viscosity and viscosity ratios is concerned, that the hydrodynamic load on the cell depends on the frequency of oscillatory shear flow as well as the amplitude of the shear stress, η_1_σ; here η_1_ is the viscosity of the outside medium and σ is the amplitude of the shear flow. Numerical algorithms work more efficiently when the ratio η_2_/η_1_ = 1 with η_2_ being the cell viscosity. For that reason simulations were often performed with η_2_/η_1_ = 1 with the understanding that has little effect as long as the amplitude of the shear stress, η_1_σ, is unchanged. This correlation has been confirmed by simulations with viscosity ratios in the range 1 ≤ η_2_/η_1_ ≤ 7. This observation is most useful when exploring a large parameter space as mentioned above. Our previous work provides much further detail as to the broader range of numerical methods (see e.g., Peng et al., 2010, 2011).

The focus herein has been on oscillatory shear flow states that produce splenic-like conditions of deformation, stress distributions around the cell, and their respective time scales. It would be of interest, however, to more systematically explore a wider range of scenarios involving various conditions of frequency, shear rate amplitude, and cell/medium viscosity. In this manner we may explore the potential effects of time scales that may allow, for example, skeletal restructuring to occur. This is a large, yet interesting, topic of future research.

The scenarios presented just above, along with all the results and analysis of section 4 support the view that vesiculation can be driven by either metabolically, or mechanically (i.e., imposed deformation), induced energetics. In either case a reduction in skeletal/bilayer binding, (i.e., aging induced reductions in γ), although not strictly required, are strongly causative influences that promote vesiculation. This view is consistent with our earlier conclusions in Zhu et al. (2017). Purely mechanically driven vesiculation is unlikely under physiological conditions since such large separating stresses are unlikely to develop. Exceptions are, for example, flow in aspiration as demonstrated by Peng et al. (2010), where we have noted the time scales as well as the severe deformations were sufficient to effectively reduce γ by dynamically restructuring the skeleton that reduces the areal density of attachment points. However, such restructuring does not occur during splenic flow or our oscillatory flow introduced herein (Zhu et al., 2017) due to the inherently shorter time scales in these types of flow. Indeed, our modeling described in section 3 defined a key parameter, ℓ_0_, that represented a membrane/skeleton area damaged by metabolic and/or biochemical means that played a strong role, if not mediated, the vesiculation process. The importance of such damaged membrane zones has been recently emphasized (Leal et al., 2018).

This view is consistent with the fact that although a given RBC transits the spleen on the order of 40 times per day, such cells only produce about 2.71 vesicles per day (Ciana et al., 2017b). *Hence vesiculation, even under conditions of large deformations, should be viewed as a rather improbable event!* An efficient way to generate vesicles systematically in laboratory conditions is to process vast number of cells simultaneously, which happens to be the advantage of our oscillatory flow device.

We have noted that the prime driving force for vesiculation in our model is ϵ_0_, the skeleton's elastic energy, may be augmented by −Δ*G*_*a*→*s*_, or at least by some part of it. This free energy is released upon a transition to a more symmetric lipid leaflet composition. But on the one hand, the kinetics for lipid flipping is not normally large and thus times scales are a question. Yet, as inner leaflet lipids like PS are exposed on vesicles, it appears that flipping does indeed occur during vesiculation; it is possible that the normally slow kinetics of lipid flipping is increased by membrane deformation. But this will alter the membrane energetics due to, e.g., non-local bending energy as well as through the pre-curvature energy term (Miao et al., 1994; Waugh, 1996; Lim et al., 2002; Mukhopadhyay et al., 2002; Bozic, et al.); specifically, it relates to, for example, how one assesses the terms involving Δ*A*_0_ in the cited works. Hence, this question awaits future detailed study.

It was noted that RBCs of those affected by Scott's syndrome display a decreased rate of vesiculation (Bevers and Williamson, 2016; Bevers et al., 2017). Indeed, Rosing et al. (1985) reported that the syndrome originates from a defective scramblase activity that suppresses PS exposure on the outer membrane leaflet. Hence these observations may support the idea that a loss in lipid asymmetry may play a direct role in promoting vesiculation as suggested above and in section 4.2.

We add that our approach allows for a thorough study of the effects of not only cytosol viscosity but also medium, or plasma, viscosity that will effect the forces and deformations experienced by the cell during a given imposed flow (Williams and Morris, 1980; Chien, 1987; Tuvia et al., 1997; Pozrikidis, 2005; Freund et al., 2012; Freund, 2013; Zhu et al., 2017). Effects of altered viscosity, including plasma viscosity, have been associated with a variety of dysfunctions and disease (e.g., Késmárky et al., 2008; Sapmaz et al., 2011; Toprak et al., 2012). In general, with increased plasma viscosity deformations, and the associated ϵ_0_, will be augmented and hence so will the prospect for vesiculation. Hence our approach offers novel prospects for pursuing such lines of future inquiry.

We herein introduced a novel theoretical/simulation approach to subject cells to *tailored* shear deformations that, mimic, and expand on the types of deformations that promote vesiculation (Zhu et al., 2017). This clearly suggests an experimental plan, based on these findings, that would subject erythrocytes to biochemical stress including, e.g., oxidative, nitrite (NOS), and Ca^2+^ uptake stress, *inter alia*, so as to induce “aging damage,” e.g., disruptions to the cell's skeleton/bilayer membrane and to follow the resulting course of promoted vesiculation as influenced by carefully tailored modes of deformation with varying shapes and intensity. The findings presented in sections 3.1 and 4 demonstrate that such an approach is viable and would clearly provide novel insights into the aging/vesiculation process.

Finally we add that, aside from the existence of non-biological walls that typically are part of flow chambers, our methods involve no artificial structures and naturally subject large numbers of cells to our tailored deformations. We demonstrate how splenic flow induced deformations can be produced and thereby provide conditions to study events such as age induced vesiculation. Our methods may also provide a valuable compliment observations made within micro fluidic devices (e.g., Deplaine et al., 2011; Picot et al., 2015) provided these can be fabricated with splenic-like slits with dimensions ≤ 1.5μm and through which red cells can actually flow through and not simply become “jammed”; such behavior involves deformations and time scales quite unlike splenic flow and is hence nonrepresentative. Moreover, although of use in visualizing individual passage through even sub-micron artificial slits (Gambhire et al., 2017), micro-fluidic chambers do not naturally deal with the very large number of cells required for vital statistical studies; recall that, *in-vivo*, vesiculation is a quite rare event, occurring roughly 1 in every 15 splenic passages.

## Author contributions

RA and QZ wrote the manuscript and performed the analysis and simulations. PC contributed to the experimental portions of the research.

### Conflict of interest statement

The authors declare that the research was conducted in the absence of any commercial or financial relationships that could be construed as a potential conflict of interest.

## Appendix

### Mathematical formulation for fluid-cell interactions

The fluid-cell interaction is mathematically formulated within a low-Reynolds number Stokes/Oseen flow (Pozrikidis, 1992) so that at any point **x**_0_ on the surface of the cell (**x**_0_ ∈ Γ_*c*_) the velocity **v** is given as

(A1) $$\begin{array}{l} \text{v}({\text{x}}_{0})=\frac{2}{1+\Lambda }\bar{\text{v}}({\text{x}}_{0})\\ \;-\frac{1}{4\pi \eta_{1}(\Lambda +1)}\iint_{\Gamma_{c}}\text{G}(\text{x},{\text{x}}_{0})\cdot \Delta \text{t}(\text{x})d\Gamma (\text{x})\\ \;+{\frac{1-\Lambda }{4\pi (1+\Lambda )}}_{\Gamma_{c}}\text{v}(\text{x})\cdot \text{T}(\text{x},{\text{x}}_{0})\cdot \text{n}(\text{x})d\Gamma (\text{x}), \end{array}$$

where $\bar{\text{v}}$ is the undisturbed flow velocity. The viscosity ratio Λ ≡ η_2_/η_1_ (η_1_ and η_2_ are the viscosities of the external fluid and the internal fluid, respectively). Δ**t** is the difference between the traction in the outside surface of the cell membrane and the traction in the inside surface of the membrane. denotes the principal value integration. The matrix **G** contains the Green's function for velocity *G*_*ij*_, and the matrix **T** contains the Green's function for stress *T*_*ijk*_. We have

(A2) $$G_{ij}(\text{x},{\text{x}}_{0})=\frac{\delta_{ij}}{\left|\text{x}-{\text{x}}_{0}\right|}+\frac{\left(x_{i}-x_{0_{i}})(x_{j}-x_{0_{j}}\right)}{|\text{x}-{\text{x}}_{0}{|}^{3}},$$

and

(A3) $$T_{ijk}(\text{x},{\text{x}}_{0})=-6\frac{\left(x_{i}-x_{0_{i}})(x_{j}-x_{0_{j}})(x_{k}-x_{0_{k}}\right)}{|\text{x}-{\text{x}}_{0}{|}^{5}},$$

where δ_*ij*_ is Kronecker's delta.

In this study $\bar{\text{v}}$ is chosen to be a linear shear flow with zero velocity at the centroid of the cell. The shear rate of this flow varies sinusoidally in time with amplitude σ. Numerically, Equation A1 is solved with a boundary-element approach (Peng et al., 2011). By discretizing the cell surface into quadruple elements, Equation A1 is rewritten as

(A4) $$\text{v}={\bar{\text{v}}}_{c}-{\text{S}}_{cc}{\text{q}}_{c}+{\text{D}}_{cc}\text{v},$$

where the global velocity vector v includes velocities at all collocation points on the cell surface and ${\bar{v}}_{c}$ contains the undisturbed velocities at these points. The matrices S_*cc*_ and D_*cc*_ are influence matrices representing the second term and the third term on the righthand side of Equation A1, respectively. In general cases, an iterative algorithm is applied to solve Equation A1. In the special case when Λ = 1, however, the term D_*cc*_v vanishes so that no iteration is needed and the computational efficiency is greatly enhanced.

We use 6,000 boundary elements on the cell surface, which guarantee numerical stability and accuracy even in cases with extreme cell deformations such as infolding (Peng and Zhu, 2013; Salehyar and Zhu, 2016; Zhu et al., 2017) (indeed, in most cases a coarser mesh with 2,000 elements is sufficient for accuracy but it may lead to instability in scenarios with large membrane curvature). With a time step of 3 × 10^−6^ second it takes a few hours to run the time integration up to 1 second in a 2.4 GHz workstation with 8 processors.

A modified version of Equation A1 (Zhao et al., 2010) was applied to solve the splenic flow problem demonstrated in Figure 3A. That version involves solid boundaries, where no-slip/no-flux boundary conditions are imposed. Besides, the configuration shown in Figure 3A is periodic in all three directions so that different forms of Green's functions (*G*_*ij*_ and *T*_*ijk*_) are used (Salehyar and Zhu, 2016).
